# Meta‐analysis of ecosystem services associated with oyster restoration

**DOI:** 10.1111/cobi.13966

**Published:** 2022-09-08

**Authors:** Rachel S. Smith, Selina L. Cheng, Max C. N. Castorani

**Affiliations:** ^1^ Department of Environmental Sciences University of Virginia Charlottesville Virginia USA

**Keywords:** fisheries, foundation species, habitat provisioning, nutrient cycling, oyster reefs, Aprovisionamiento de hábitat, bancos de ostión, ciclo de nutrientes, especies fundadoras, pesquerías

## Abstract

Restoration of foundation species promises to reverse environmental degradation and return lost ecosystem services, but a lack of standardized evaluation across projects limits understanding of recovery, especially in marine systems. Oyster reefs are restored to reverse massive global declines and reclaim valuable ecosystem services, but the success of these projects has not been systematically and comprehensively quantified. We synthesized data on ecosystem services associated with oyster restoration from 245 pairs of restored and degraded reefs and 136 pairs of restored and reference reefs across 3500 km of U.S. Gulf of Mexico and Atlantic coastlines. On average, restoration was associated with a 21‐fold increase in oyster production (mean log response ratio = 3.08 [95% confidence interval: 2.58–3.58]), 34–97% enhancement of habitat provisioning (mean community abundance = 0.51 [0.41–0.61], mean richness = 0.29 [0.19–0.39], and mean biomass = 0.69 [0.39–0.99]), 54% more nitrogen removal (mean = 0.43 [0.13–0.73]), and 89–95% greater sediment nutrients (mean = 0.67 [0.27–1.07]) and organic matter (mean = 0.64 [0.44–0.84]) relative to degraded habitats. Moreover, restored reefs matched reference reefs for these ecosystem services. Our results support the continued and expanded use of oyster restoration to enhance ecosystem services of degraded coastal systems and match many functions provided by reference reefs.

## Abstract 2

*Article Impact Statement*:Restored oyster reefs enhance ecosystem services relative to degraded habitats and match those of references, supporting future restoration.

## Resumen

La restauración de especies fundadoras promete revertir la degradación ambiental y restituir servicios ambientales perdidos, pero la falta de evaluación estandarizada de proyectos limita la comprensión de la recuperación, especialmente en sistemas marinos. Los bancos de ostión son restaurados para revertir declinaciones globales masivas y recuperar servicios ecosistémicos valiosos, pero el éxito de estos proyectos no ha sido cuantificado sistemática ni integralmente. Sintetizamos datos sobre los servicios ecosistémicos asociados con la restauración de ostiones de 245 pares de bancos restaurados y degradados y 136 pares de bancos restaurados y de referencia a lo largo de 3500 km de costa del Golfo de México y Atlántico norteamericanos. En promedio, la restauración se asoció con un incremento de 21 veces en la producción de ostión (media de proporción de respuesta log = 3.08 [95% IC 2.58‐3.58]), mejoras entre 34 y 97% en el aprovisionamiento de hábitat (abundancia media = 0.51 [0.41‐0.61], riqueza media = 0.29 [0.19‐0.39], y biomasa media = 0.69 [0.39‐0.99]), 54% más remoción de nitrógeno (media = 0.43 [0.13‐0.73]), y 89–95% más nutrientes en sedimento (media = 0.67 [0.27‐1.07]) y materia orgánica (media = 0.64 [0.44‐0.84]) en relación con hábitats degradados. Más aun, estos servicios ecosistémicos de los bancos restaurados fueron muy similares en los bancos de referencia. Nuestros resultados sustentan el uso continuo y expandido de la restauración de ostiones para mejorar los servicios ecosistémicos de sistemas costeros degradados y que sean parecidos a las numerosas funciones proporcionadas por los bancos de referencia.

## INTRODUCTION

Environmental degradation associated with human activities has caused worldwide declines of foundation species (Ellison, 2019; Ellison et al., 2005) and associated losses of their ecosystem services—the benefits and uses that humans obtain from ecosystems (Barbier et al., 2011; Millennium Ecosystem Assessment, 2005). Restoration promises to recover lost ecosystem services and approximate the attributes of target reference systems (Jones et al., 2018; Miller & Hobbs, 2007). Despite a multibillion dollar restoration economy (BenDor et al., 2015; Kimball et al., 2015) and a global call to expand restoration during the United Nation's Decade of Restoration (2021−2030) (Cooke et al., 2019), restoration science remains a relatively young field that has only recently gained enough empirical studies to examine generalizable patterns across projects (Cooke et al., 2019; Wortley et al., 2013). For example, new synthesis studies have systematically quantified enhancement of many ecosystem services following restoration of freshwater and terrestrial systems (Benayas et al., 2009; Crouzeilles et al., 2016; Meli et al., 2014; Miller et al., 2010; Shimamoto et al., 2018). However, similar generalizations about the effects of restoration across multiple ecosystem services are rare for many marine systems (Abelson et al., 2016; but see Orth et al., 2020; Su et al., 2021), despite widespread declines of valuable species that form the foundation of these ecosystems (Kirby, 2004; Pandolfi et al., 2003; Polidoro et al., 2010; Waycott et al., 2009) and substantial investment in restoration projects to counter these losses (Bayraktarov et al., 2016; Duarte et al., 2020).

The loss of oyster populations due to overfishing and disease is one of the starkest declines of foundation species worldwide; over 85% of global oyster reefs have been lost since the 1800s (Beck et al., 2011). To counter these losses, oyster restoration projects have increased exponentially since 1990 across Asia, Australia, Europe, and North America (Duarte et al., 2020). Oyster restoration aims to enhance or establish reefs by adding hard substrate or live oysters to the seafloor in soft‐sediment coastal environments (Bersoza Hernández et al., 2018). In theory, substrate addition attracts larval oysters from existing populations to gregariously settle and build new reefs, whereas live oyster additions provide brood stock to increase larval supply and enhance recruitment (Brumbaugh & Coen, 2009; Lipcius et al., 2008). In practice, however, such restoration efforts have mixed evidence of success (Geraldi et al., 2013; Kennedy et al., 2011; Powers et al., 2009; Schulte et al., 2009; Smith et al., 2005), despite substantial financial investments by governments and nonprofit organizations (e.g., America Recovery and Reinvestment Act of 2009—$167 million [Samone et al., 2017]; U.S. RESTORE Act—$133.3 million [Gulf Coast Ecosystem Restoration Council, 2019]; Australia's Reef Builder project—$20 million [The Nature Conservancy Australia, 2020]). Critical, standardized evaluation is needed to determine the extent to which oyster restoration achieves desired outcomes across projects and different ecosystem services.

Although early oyster restoration efforts focused on recovering wild oyster fisheries for harvest, recent shifts toward ecosystem‐based management instead target reclaiming ecosystem services associated with oyster reefs (Coen et al., 2007; Grabowski et al., 2012). Valued ecosystem services on oyster reefs include oyster production (e.g., oyster abundance, density, biomass), habitat provisioning for fishes and invertebrates, water filtration, biogeochemical cycling, shoreline protection, and carbon storage (Coen et al., 2007; Grabowski et al., 2012). Excluding oyster harvest, the estimated value of these ecosystem services ranges widely from $5500 to $99,000∙ha^–1^∙year^–1^, depending on reef location and what services are measured and achieved (Grabowski et al., 2012). Synthesis studies show that oyster reefs generally enhance habitat provisioning (Peterson et al., 2003; zu Ermgassen et al., 2016; La Peyre et al., 2019) and biogeochemical cycling (Ray & Fulweiler, 2021) relative to bare sediment, but these studies grouped restored and natural oyster reefs together and thus did not estimate the effects of oyster restoration per se on ecosystem services. Moreover, although there is broad evidence across studies that restoration can enhance recruitment of juvenile fish and swimming crabs relative to bare sediment (reviewed in Davenport et al., 2021), a wider suite of ecosystem services on restored reefs has not been evaluated systematically to assess whether restored reefs enhance ecological functions relative to degraded reefs. Furthermore, ecosystem services have not been compared directly between restored reefs and natural reference reefs that serve as restoration targets.

To address these gaps, we conducted a meta‐analysis to systematically quantify the success and uncertainty of oyster reef restoration for a suite of biological, biogeochemical, and physical ecosystem services relative to both degraded and natural reference habitats. We focused on the eastern oyster (*Crassostrea virginica*), the oyster species native to the East Coast of the United States. Like other oyster species, the extent and biomass of eastern oyster populations have declined dramatically since the 1800s due to overfishing and disease (Kirby, 2004; zu Ermgassen, Spalding, Blake, et al., 2012). Most oyster restoration projects have targeted this species (Duarte et al., 2020). About 4.5% of historically lost reefs in the United States have been restored (1768 projects, 5199 ha restored, $299,999/ha) (Bersoza Hernández et al., 2018), and these projects provide a robust sample size for meta‐analysis of restored reefs and control areas. To evaluate whether restored eastern oyster reefs enhance ecosystem services relative to degraded habitats and whether restored reefs provide ecosystem services equivalent to reference reefs, we synthesized data from 245 restored–degraded reef pairs and 136 restored–reference reef pairs from 106 publications collected along 3500 km of the U.S. Gulf of Mexico and Atlantic coastlines. Our findings reveal the strength and variability of effects of oyster restoration on a suite of ecosystem services, including oyster production, habitat provisioning, biogeochemical cycling, water clarity, and shoreline protection.

## METHODS

### Definitions

**FIGURE 1:**
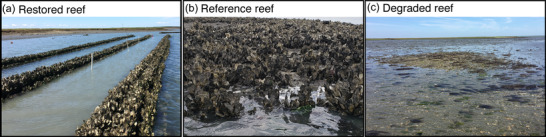
Examples of a (a) restored reef created to resemble the structure and function of natural reefs, (b) reference reef that represents the desired end point of restoration, and (c) degraded reef that represents the starting point of restoration or an undesirable end point. Photos by Kinsey Tedford and Bo Lusk

We defined restored reefs as artificial or experimental reefs created to establish (or reestablish) habitat resembling the structure and function of a natural reef (Figure 1a). Restored reefs included any addition of substrate (e.g., shell, concrete, limestone, rock) or live oysters to create patch or fringing reefs in intertidal or subtidal coastal areas. Restored reefs were constructed from 1940 to 2018 and sampled at intervals ranging from 1 day to 62 years postconstruction (median 24 months). Natural reefs with pronounced vertical structure or live oyster densities across multiple size classes served as reference reefs to represent the desired end point of restoration (Figure 1b). Unaltered areas (bare or unstructured sediment) served as degraded reefs that represented restoration starting points or undesired endpoints (Figure 1c). Defining restoration targets is challenging in systems that are severely degraded and where historic population levels are likely unattainable (Suding, 2011). However, most restoration projects aim to build reefs that can match the attributes of natural remnant reefs, which vary in quality by location. We used the publications’ descriptions of comparison reefs to determine reef classification and contacted authors to clarify this distinction when unclear (40 of 106 papers required clarification).

We characterized the effects of reef restoration on 5 ecosystem services: oyster production, habitat provisioning, biogeochemical processes, water clarity, and shoreline protection. Oyster production included all measures of oyster abundance (i.e., density, count, percent cover, biomass). We included all oyster measures in the broad grouping total oysters. Where data were available, we also categorized oysters by shell height (adults: ≥76 mm; juveniles: ≤75 mm) (Harding et al., 2008). Where oyster life stage was described without size measurement, we assumed juveniles were ≤75 mm and adults were ≥76 mm. When reported, we also collected environmental responses commonly used in habitat suitability models to choose reef restoration sites, including temperature, salinity, and dissolved oxygen.

Habitat‐provisioning responses included measures of taxon‐specific abundance (i.e., density, count, percent cover), taxon‐specific biomass, taxa richness (hereafter, richness), and length of individual organisms. If both density and count were measured for a given taxon, we only included density. We grouped taxon‐specific abundances by habitat‐use group: nekton (free‐swimming fishes and crustaceans), epifaunal invertebrates (mobile and sessile species dwelling on reef surfaces and interstices), infaunal invertebrates (collected in sediment cores), birds, and plants (seagrasses, salt marsh plants). These groupings correspond with species habitat use and match categories commonly used in the studies (Gittman et al., 2016). We further classified species by order and family for common taxonomic groups of fishes and invertebrates, extracting taxonomic hierarchies with the taxize package (Chamberlain & Szöcs, 2013) in R 3.5.2, which we used for all analyses (R Core Team, 2022).

We estimated the effect of reef restoration on several key biogeochemical processes, including sediment nitrogen removal and concentrations of nutrients, organic matter, and chlorophyll in the sediment and water column. Nitrogen removal responses included measures of net N_2_ flux, actual denitrification, and potential denitrification taken with sediment cores and in situ incubation chambers. Nutrient responses included measures of nitrate, ammonium, total nitrogen, phosphate, total phosphate, and soluble reactive phosphorus.

Water clarity was represented by measures of total suspended sediments, turbidity, light attenuation, and Secchi depth. We inverted the values of total suspended sediments and turbidity to standardize the direction and biological interpretation across water clarity responses.

For shoreline protection responses, we divided the hydrodynamic changes associated with reef structure into dampening water flow or wave height around reefs and changing shoreline movement proximate to reefs. To standardize across studies and remove negative values from the log response ratio (LRR) calculations, we converted all measures of shoreline movement to shoreline advance (i.e., seaward movement of the shoreline).

### Literature search and inclusion criteria

We followed the Preferred Reporting Items for Systematic Reviews and Meta‐Analyses (PRISMA) standards for meta‐analysis reporting (Moher et al., 2009) (Appendices S1 & S2).

We performed 2 searches on Web of Science in the Science Core Collection to identify candidate publications (search date 17 October 2019). In the first search, we used the search string: oyster definition (“*Eastern oyster*” OR “*Crassostrea virginica*” OR “oyster reef*”) AND ecological response (*richness* OR *diversity* OR *abundance* OR densit* OR *cover* OR *growth* OR *fitness* OR “ecosystem service*” OR “ecosystem function*” OR “ecosystem process*” OR *habitat* OR *environment* OR *ecosystem* OR *substrate* OR *production* OR *biomass* OR *number* OR *frequency* OR *similarity* OR *filtration* OR *nutrient* OR *sediment* OR flux*) AND restoration indicator (restor* OR re‐creat* OR rehabilitat* OR recover* OR *mitigation*).

The second search used the same string as above, but we replaced the restoration indicator keywords with (reef*) to capture studies on artificial or experimental reefs that met our definition of restored reefs. To collect recent papers excluded in the Web of Science search, we used these 2 search strings to run another search in Google Scholar for papers published from 2019 to 2020 (search date: January 26, 2020). We identified additional papers from in‐text citations, Google Scholar search‐term alerts, existing reviews of ecosystem services on restored oyster reefs (Davenport et al., 2021; La Peyre et al., 2019; Ray & Fulweiler, 2021; zu Ermgassen et al., 2016), and communications with publication authors.

For inclusion, publications had to contain an experimental or observational field study of the eastern oyster in which the authors collected data for at least 1 of the ecosystem services described above on both a restored reef and on either a reference reef or a degraded reef. We screened the title, keywords, and abstracts of 1121 candidate publications and identified 106 for inclusion (Appendices S1, S3, & S4).

### Data extraction

For each publication, we extracted means (or sums), sample sizes, and standard deviations (when reported) for each ecological response measured on each restored reef and its associated reference or degraded reef. We averaged responses across replicate samples for each reef. If the authors did not explicitly match a restored reef with a reference or degraded reef, we chose the nearest reference or degraded reef to each restored reef to minimize variability in local physical and abiotic conditions. When a response was measured repeatedly over time at an individual reef, we used data only from the final measurement to avoid temporal autocorrelation (Koricheva et al., 2013) and maximize time since construction of the restored reef. If the same response variable was presented for multiple restored reefs and as a spatial average (across all restored reefs), we used the individual responses for each restored reef. We collected data from the publication text and tables and used the metaDigitise package to extract data from figures (Pick et al., 2019). We also collected metadata for geographic location, reef construction date, sampling date, and ecological response type with its units.

### Calculating effect sizes

We used the LRR to measure the proportional change of an ecosystem service on restored oyster reefs relative to either degraded reefs or reference reefs (LRR = ln[value on restored reef/value on comparison reef]) (Koricheva et al., 2013). An effect size of 0 indicated no difference in ecological response between the restored reef and the comparison reef; positive values indicated greater responses on the restored reef and negative values indicated lesser measures on the restored reef. We calculated LRR because measures of variation were not available or estimable for many of the ecological responses. Furthermore, accounting for variance in meta‐analysis models may not substantially alter outcomes in model simulations (Song et al., 2020). Nevertheless, we included additional effect size calculations that incorporated variance in the supplement, including Hedges’ *d* and LRRs weighted by the inverse of the variance and sample size (Appendices S8 & S19–S23). We removed effect sizes where the mean values of restored and degraded or reference reefs were both 0 (Koricheva et al., 2013). If the mean value of only 1 of the responses was 0, we added the minimum value that was likely to be detected with the associated sampling method to both responses (e.g., a count of 1 for number of individuals per quadrat) (Poore et al., 2012). To include negative responses in LRRs, we shifted values for both paired responses by the absolute value of the most negative response plus 1 to maintain the difference between responses. Finally, we calculated the percent change between restored reefs and comparison reefs as 100 × e^(LRR – 1)^ (Pustejovsky, 2018).

### Statistical analyses

We calculated the mean LRR and 95% confidence intervals (CIs) for each response category described above for degraded and reference reefs. To test whether the LRRs for each category differed from 0, we used the glmmTMB package to create a linear mixed model (LMM) with publication as a random intercept to account for within‐study correlation (Brooks et al., 2017). We excluded ecological responses included in fewer than 3 publications for oyster production and habitat provisioning variables and in fewer than 2 publications for the remaining ecosystem functions, which were more data limited. We used the DHARMa package for LMM regression diagnostics to ensure that model residuals met assumptions of normality and homogeneity (Hartig, 2019). Spline correlograms of model residuals showed no evidence of spatial autocorrelation (Zuur et al., 2009). Our analyses included multiple statistical tests (70 independent ecological responses), which increased the possibility of erroneously rejecting at least 1 null hypothesis (type I error). To aid interpretation, we report raw *p*‐values and their associated sample sizes and mean effect sizes and their corresponding 95% CIs. Given the number of tests and possibility for type I errors, *p*‐values close to α = 0.05 should be interpreted cautiously, particularly in cases where the sample size is small (<5 papers) (Althouse, 2016; Feise, 2002).

To assess the potential for publication bias to overrepresent significant results in the meta‐analysis, we assessed funnel plots of effect sizes versus sample size for asymmetry (Møller & Jennions, 2001); calculated Rosenthal's fail‐safe number to indicate the number of nonsignificant, unpublished studies needed to remove a significant overall effect size (Rosenthal, 1979); and used a drop‐one approach to assess whether any study exerted undue influence on the overall mean LRR (Lefcheck et al., 2019). These analyses suggested that our findings are robust to publication bias (Appendices S8–S11). To assess how the abundance and proportion of included studies changed from 1995 to 2020, we fitted 2 separate linear models with the number and proportion of included studies as a function of publication year.

## RESULTS

### Temporal and geographic trends in studies of oyster reef restoration

We analyzed 106 studies (88 peer‐reviewed articles, 11 theses, and 7 reports) published from 1960 to 2020, yielding *n* = 4093 individual effect sizes. Of these effect sizes, 68% compared restored reefs with degraded reefs that represented the starting point of restoration or undesirable endpoints (2780 effect sizes from 245 restored–degraded reef pairs) and 32% compared restored reefs to reference reefs that represented restoration targets (1313 effect sizes from 136 restored–reference reef pairs).

**FIGURE 2:**
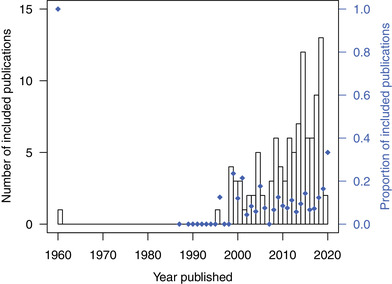
The number of included publications (bars) and proportion of publications (points) published annually in a meta‐analysis of ecosystem services on restored reefs relative to degraded and reference reefs. Years without points indicate years with no publications (included or not included)

In assessing studies for inclusion, most studies published before 1999 did not measure ecological responses on both restored and comparison reefs or inclusion criteria were otherwise not met (Figure 2; Appendix S3). Indeed, we excluded 54 studies in which ecosystem services on restored reefs were measured but comparable measures on degraded or reference reefs were not included (Appendix S3). The number of studies that met our criteria increased sharply beginning in the late 1990s and rose steadily through at least 2019 (*F*_1,24_ = 30.8, *p* < 0.0001) (Figure 2). This change likely reflected a gradual increase in the total number of restoration studies (included + not included) because the average proportion of included studies was relatively constant at ∼10% from 1995 to 2020 (*F*_1,24_ = 2.7, *p* = 0.12) (Figure 2).

**FIGURE 3:**
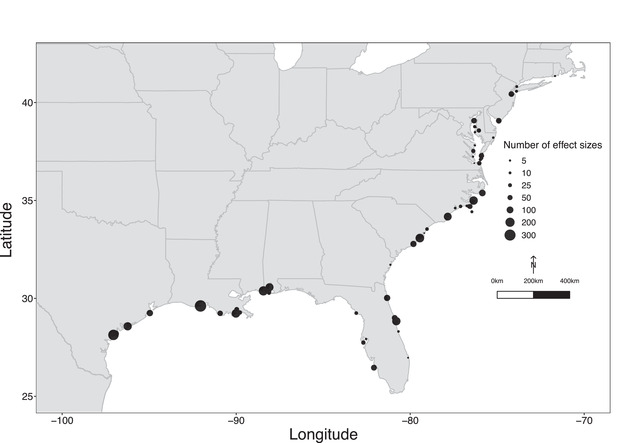
Distribution across the U.S. Gulf of Mexico and Atlantic coasts of the 4093 effect sizes collected from 106 publications about ecosystem services on restored oyster reefs and associated degraded and reference reefs (points, locations of studies included in the meta‐analysis; the larger the point, the greater the effect size)

Most effect sizes came from studies conducted along the U.S. Gulf of Mexico (especially between Corpus Christi Bay and Mobile Bay), Mid‐Atlantic, and southern Atlantic coasts. Few studies from northern Florida, Georgia, and the Northeast (Figure 3) could be included, despite these areas supporting substantial oyster populations and oyster restoration projects (Dunnigan, 2015; Frederick et al., 2016; Locher et al., 2021; Taubenheim, 2015; Taylor & Bushek, 2008).

We found no differences between restored reefs and degraded or reference reefs in abiotic factors known to influence oyster survival and growth, including temperature, salinity, and dissolved oxygen (*p* > 0.05) (Appendix S7), suggesting that restored reefs are generally sited in areas with suitable water conditions for oysters.

For all ecosystem services, within‐study variance was consistently low (Appendices S12–S18). When we repeated our analysis with LRRs weighted by the inverse of the variance or the sample size (Appendices S19–S23), we found comparable results to the main analysis for all ecosystem services. However, significant effects for habitat provisioning responses (abundance, biomass, richness, or length) were lost when we repeated the analysis with Hedges’ *d*, which excluded 57% of effect sizes because the studies did not report sample variances (Appendices S8 & S20).

### Oyster production

**FIGURE 4:**
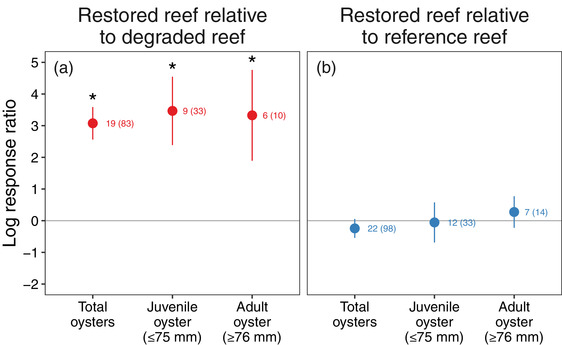
Mean log response ratios for oyster abundance on restored reefs relative to (a) degraded reefs (red) (value > 0, increase in oyster abundance on restored reefs relative to paired degraded reefs) and (b) reference reefs (blue) (values near 0, similar abundances on paired restored and reference reefs) across all oyster size classes (bars, 95% CIs; asterisks, effect sizes that differ from 0 at *p* ≤ 0.05). Total oysters include effect sizes for juvenile and adult oysters and from studies that reported pooled abundances of juvenile and adult oysters. The number of publications and effect sizes (in parentheses) are shown next to each mean effect size

On average restoration greatly increased oyster production compared with degraded areas (Figure 4a) and approximated that of reference reefs (Figure 4b). Relative to degraded reefs, restored reefs supported 31 times more juvenile oysters (*F*_1,24_ = 6.3, *p* = 0.02), 27 times more adult oysters (*F*_1,16_ = 5.7, *p* = 0.05), and 21 times more total oysters, which included all effect sizes for juvenile and adult oysters, as well as effect sizes for pooled abundances of juveniles and adults (*F*_1,64_ = 30.0, *p* < 0.001). Oyster densities were similar on restored reefs and reference reefs for juveniles (*F*_1,21_ = 0.5, *p* = 0.5), adults (*F*_1,7_ = 1.2, *p* = 0.3), and total oysters (*F*_1,76_ = 1.6, *p* = 0.2).

### Provisioning habitat and supporting reef taxa

**FIGURE 5:**
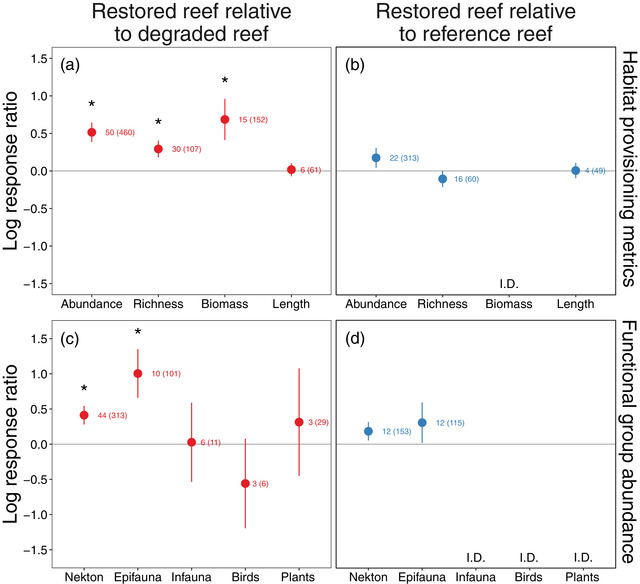
Mean log response ratios for habitat provisioning metrics on restored reefs relative to (a) degraded reefs and (b) reference reefs and functional group abundance on restored reefs relative to (c) degraded reefs and (d) reference reefs (colors, points, error bars, asterisks, and numbers as in Figure 4; I.D., insufficient data for analyses [<3 publications])

Restoration generally enhanced reef communities relative to degraded habitats (Figure 5a) and matched reference reefs (Figure 5b). Restored reefs had 67% greater total community abundance (*F*_1,410_ = 14.9, *p* < 0.001), 34% greater taxonomic richness (*F*_1,77_ = 14.7, *p* < 0.001), and 99% greater total community biomass (*F*_1,13_ = 5.8, *p* = 0.02) than degraded reefs (Figure 5a). Moreover, restored reefs supported reef communities similar in abundance (*F*_1,291_ = 1.5, *p* = 0.2) and richness (*F*_1,44_ = 0.6, *p* = 0.5) to reference reefs (Figure 5b). There were not enough studies of community biomass on restored and reference reefs to examine this comparison (2 studies). Organisms on restored reefs were similar in length to those on degraded reefs (*F*_1,55_ = 0.2, *p* = 0.6) and reference reefs (*F*_1,45_ < 0.01, *p* = 0.9), suggesting no effect of restoration on the size of reef taxa.

However, restoration‐driven enhancements of reef communities varied among taxa functional groups (Figure 5c, d). For studies reporting faunal abundances by functional group, restoration increased nekton abundance by 51% (*F*_1,269_ = 12.8, *p* < 0.001) and epifauna abundance by 173% (*F*_1,91_ = 16.2, *p* < 0.001) relative to degraded reefs (Figure 5c). Moreover, there was no difference between restored and reference reefs for nekton (*F*_1,141_ = 0.3, *p* = 0.6) or epifauna (*F*_1,103_ = 0.5, *p* = 0.5) (Figure 5d). By contrast, abundances of infauna (*F*_1,6_ = 0.2, *p* = 0.7), bird (*F*_1,3_ = 0.06, *p* = 0.8), and plants (*F*_1,26_ = 0.07, *p* = 0.8) were similar on restored and degraded reefs (Figure 5c). Few studies measured infauna, birds, or plants on paired restored and reference reefs (infauna, 2 studies; birds, 2 studies; plants, 1 study), so it is unclear whether restoration failed to recover these groups or whether they were naturally similar between restored and reference reefs (Figure 5d).

For studies reporting finer‐scale taxonomic data, toadfishes, clingfishes, mullets, eels, and perch‐like fishes were more abundant on restored reefs than degraded reefs (Appendix S5). Common crabs (including stone, mud, and swimming crabs) and shrimps (e.g., snapping, palaemonid, and penaeid shrimps) were also more abundant on restored reefs than degraded reefs (Appendix S5). Abundances of most fish, crab, and shrimp taxa were similar between restored and reference reefs, although clingfishes were less abundant on restored reefs relative to reference reefs and mullets and palaemonid shrimps were slightly more abundant on restored reefs than reference reefs (Appendix S6).

### Other ecosystem functions

**FIGURE 6:**
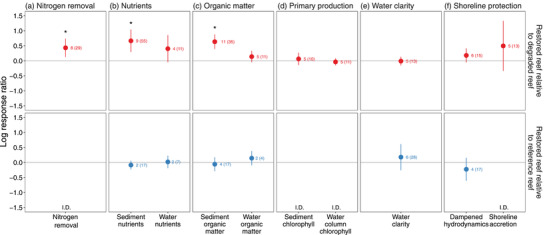
Mean log response ratios for (a) nitrogen removal, (b) sediment and water column nutrients, (c) sediment and water column organic matter, (d) sediment and water column chlorophyll, (e) water clarity, and (f) dampened water flow and shoreline advance for restored relative to degraded reefs (top panels) and for restored relative to reference reefs (bottom panels) (colors, points, error bars, asterisks, and numbers as in Figure 4; I.D., insufficient data for analyses [<2 publications])

Detectable benefits of oyster reef restoration to other ecosystem functions were limited to sediment nutrient cycling and sediment organic matter (Figure 6). Restoration enhanced nitrogen removal by 54% (*F*_1,21_ = 4.36, *p* = 0.04) (Figure 6a, top), nearly doubled sediment nutrient concentrations (*F*_1,46_ = 4.4, *p* = 0.04) (Figure 6b, top), and increased sediment organic matter by 89% (*F*_1,24_ = 7.1, *p* = 0.01) (Figure 6c, top) relative to degraded reefs. Sediment nutrients (*F*_1,15_ = 1.4, *p* = 0.3) and sediment organic matter (*F*_1,13_ = 0.29, *p* = 0.6) on restored reefs matched those of reference reefs (Figure 6b,c, bottom). There were no studies of nitrogen removal on restored and reference reefs to quantify this comparison.

However, other measures of biogeochemical function (water column nutrients, water column organic matter, sediment, and water column chlorophyll) were similar between restored reefs and degraded reefs (*F* < 3.2, *p* > 0.1) (Figure 6b–d, top), as well as between restored and reference reefs (*F* < 0.1, *p* > 0.1) (Figure 6b–d, bottom). Similarly, restoration also had no detectable effects on water clarity and measures of shoreline protection (dampened hydrodynamics, shoreline advance) relative to degraded (*F* < 1.0, *p* > 0.1) or reference reefs (*F* < 0.2, *p* > 0.1) (Figure 6e,f). No studies measured sediment or water column chlorophyll on restored and reference reefs, and only 1 study measured shoreline advance for restored and reference reefs.

## DISCUSSION

**FIGURE 7:**
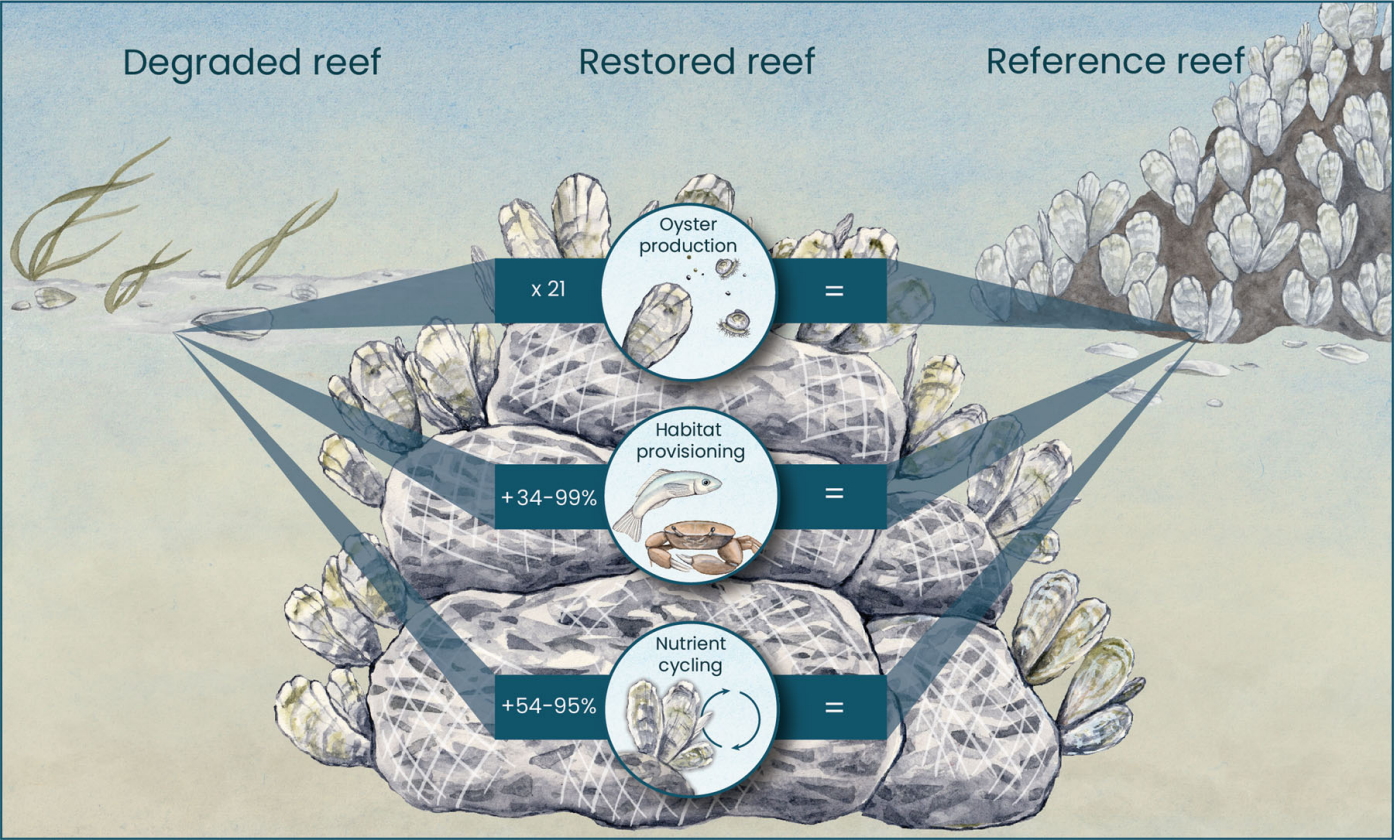
Effects of oyster reef restoration on oyster production, habitat provisioning, and nutrient cycling relative to degraded and reference reefs. Illustration by Julie Johnson, Life Science Studios

Ours is the first meta‐analysis to quantify the effects of oyster restoration on a suite of ecosystem services relative to degraded reefs and reference reefs representing restoration targets. Synthesizing across 245 restored–degraded reef pairs and 136 restored–reference reef pairs along 3500 km of U.S. coastline, we found that restoration increased oyster production by 21‐fold, biodiversity and habitat provisioning by 34–99%, and nutrient cycling by 54–95% relative to degraded habitats (Figure 7). Moreover, restored reefs matched reference reefs for these ecosystem services (Figure 7). Nevertheless, some reported benefits of oyster restoration, including increased water clarity, shoreline advance, and wave dampening, were equivocal or did not have enough available data to assess the effect of restoration relative to degraded or reference habitats; these ecosystem services warrant additional study. Despite these limitations, our results support the continued and expanded use of oyster restoration to enhance ecosystem services of degraded coastal systems and match many of those provided by reference reefs.

More broadly, these results add to growing evidence that restoration of foundation species can enhance a suite of ecosystem services. Our work supports the findings of prior syntheses that show restoration increases ecosystem services in freshwater wetlands (Meli et al., 2014), forests (Crouzeilles et al., 2016; Shimamoto et al., 2018), grasslands (Ren et al., 2016), agroecosystems (Barral et al., 2015), and other temperate and tropical freshwater and terrestrial ecosystems (Benayas et al., 2009). Our results add to synthesis studies across a range of coastal ecosystems indicating that restoration enhances populations of foundation species (Bayraktarov et al., 2016; Bosire et al., 2008; van Katwijk et al., 2015) and increases habitat provisioning (Baumann et al., 2020; Hollweg et al., 2020; Minello et al., 2003; Ning et al., 2021; Paxton et al., 2020). Our new findings demonstrate that restoring coastal foundation species can also increase nutrient cycling. Although prior syntheses have shown that marsh restoration can increase shoreline protection (Gedan et al., 2010; Shepard et al., 2011; Spalding et al., 2014), further study is needed to substantiate similar benefits from oyster restoration. Our work extends earlier findings by providing systematic assessment of multiple ecosystem services associated with oyster restoration and by making direct comparisons of restored habitats to both degraded and reference systems.

### Greater oyster production with restoration

Persistent, self‐sustaining oyster populations are necessary to support associated ecosystem services that scale with oyster biomass (Grabowski et al., 2012; zu Ermgassen et al., 2016; zu Ermgassen, Spalding, Grizzle, et al., 2012). Our results revealed that restoration typically returned oyster populations to production levels that vastly exceeded degraded reefs and also matched standing stocks of reference reefs. Furthermore, levels of enhancement were similar for juvenile (≤75 mm) and adult (≥76 mm) oysters, indicating that restored reefs generally support oyster recruits and adults that constitute brood stock. Multiple oyster size classes are essential to sustain reefs over time and their presence is a common indicator of restoration success (La Peyre et al., 2014; Schulte et al., 2009). Additionally, restoration‐driven increases in oyster biomass may support wild oyster harvest on restored reefs and in nearby areas that are demographically linked through larval dispersal (Theuerkauf et al., 2021). Substantial increases of adult and juvenile oyster densities on restored reefs support the continued use of restoration to recover foundation species that create habitat, support fisheries, and provide associated ecosystem services.

### Enhanced taxon‐specific habitat provisioning with restoration

Restoration generally increased the abundance and biomass of reef‐associated taxa relative to degraded reefs and matched those of reference reefs. Nekton and epifauna were consistently more abundant on restored reefs relative to degraded reefs, supporting prior syntheses that documented enhanced fisheries production on oyster reefs (Davenport et al., 2021; La Peyre et al., 2019; Peterson et al., 2003; zu Ermgassen et al., 2016). Several reef‐associated species were more abundant on restored reefs than degraded habitats, including toadfish, mud crabs, and snapping shrimp. Many of these fast‐growing species recruit quickly to oyster reef structure, where they gain food and refuge, while serving as prey for higher trophic levels (Grabowski et al., 2020; Smith et al., 2022; White & Wilson, 1996). Restoration also increased the abundance of many commercially valuable species, including mullets, perch‐like fishes, swimming crabs, stone crabs, and penaeid shrimps. Of the 271 taxa identified to species level in the data set, nearly half (128) are commercially fished or farmed (Food and Agriculture Organization of the United Nations, 2021), highlighting the importance of oyster reefs and their restoration to coastal economies and food security. Furthermore, 10 of these taxa are classified as vulnerable and 3 as endangered (IUCN, 2021), suggesting that restoration of oyster reefs can potentially provide habitat to threatened marine species.

In contrast to nekton and epifauna, the abundances of infauna, birds, and plants did not differ between restored and degraded habitats. This finding may be a true null result because unstructured mudflats support a diversity of infauna, serve as foraging ground for shorebirds, and provide substrate for rooted plants (Beninger, 2018). Alternatively, because fewer studies target these taxa, it is possible that limited sample size (<5 papers) provided low power to detect differences (Jackson & Turner, 2017). Furthermore, because most oyster restoration projects are monitored on short time scales (e.g., 24‐month median duration for studies in our analysis) (Bayraktarov et al., 2016), fast‐growing, mobile species, such as nekton and epifauna, may be preferentially sampled over slower colonizing infauna, birds, and plants. Thus, a third possibility could be that insufficient time had elapsed after restoration to document the return of slow‐growing or short‐dispersing species (Baumann et al., 2020; Roman & Burdick, 2012; Warren et al., 2002), although shorebirds quickly return to recently restored reefs (Frederick et al., 2016; Shaffer et al., 2019) and some temperate infauna can rapidly colonize new oyster reefs (Grabowski et al., 2005).

Restoration also increased taxonomic richness relative to degraded habitats and matched that of reference reefs. Increasing biodiversity is a common goal of restoration projects (Suding, 2011), both as a stand‐alone ecosystem service and as a proxy for other ecosystem services that are positively correlated with biodiversity (Benayas et al., 2009; Mace et al., 2012). Relative to degraded habitats, the observed 34% increase in richness with oyster restoration was comparable to levels of biodiversity enhancement documented in syntheses for forests (15–84% [Crouzeilles et al., 2016]), freshwater wetlands (15–53% [Meli et al., 2014]), salt marshes (∼30% [Ning et al., 2021]), and a range of aquatic and terrestrial habitats (44%) (Benayas et al., 2009). Although restoration may not lead to full recovery of biodiversity relative to reference habitats (Benayas et al., 2009; Crouzeilles et al., 2016) and biodiversity surveys can be incomplete measures of community structure (Martin et al., 2019; Meyer et al., 2015), our finding that taxa richness on restored reefs approximated reference reefs also supports prior work in freshwater wetlands, rocky reefs, and coral reefs (Meli et al., 2014; Paxton et al., 2020).

Although several of the significant effects for habitat provisioning responses were lost when we repeated the analysis with Hedges’ *d* (which excluded more than 50% of the collected effect sizes due to missing sample variances), our findings are consistent with the findings of prior syntheses that also showed enhanced habitat provisioning with restoration relative to unstructured habitats (Davenport et al., 2021; La Peyre et al., 2019; Peterson et al., 2003; zu Ermgassen et al., 2016). We used LRRs so that we could include abundance and richness data, which are frequently reported without variance.

### Increased benthic nutrient cycling with restoration

We found that restoration increased benthic nitrogen removal, sediment nutrients, and sediment organic matter relative to degraded reefs. This finding bolsters the conclusion of a recent synthesis that showed general enhancement of sediment nutrient cycling on oyster reefs relative to unstructured benthic habitats (Ray & Fulweiler, 2021). As oysters feed, they remove organic matter from the water column and excrete the remaining nutrients to the surrounding sediment. These nutrients can be trapped by microphytobenthos, recycled back to the water column to support primary production, or removed from the system via microbially mediated denitrification (Kellogg et al., 2013; Newell et al., 2005). Thus, our results suggest that restoration can reclaim several aspects of nutrient cycling by oysters, including sediment nutrient deposition and nitrogen removal.

However, enhanced nutrient cycling on restored reefs did not extend to the water column, which could be due to differences in spatial scaling. Furthermore, there was no difference between sediment or water column chlorophyll on restored reefs relative to degraded habitats. Likewise, we found no detectable effects of restoration on water clarity, dampened hydrodynamics, and shoreline advance. Although restored reefs appeared to match reference reefs for water column nutrients, water column organic matter, water clarity, and dampened hydrodynamics, these results are equivocal because there was no difference between restored and degraded reefs for these services. As with the undetected effects on certain groups of organisms described above, these findings could reflect true null results (i.e., oyster restoration had no effect relative to degraded habitats), low statistical power due to a limited number of studies, or an insufficient duration of time elapsed following restoration (particularly in the case of shoreline movement measurements). It is also possible that some restoration projects were too small to exert detectable effects on certain ecosystem functions, such as shoreline protection or biogeochemical attributes of the water column (Morris et al., 2019; Pomeroy et al., 2006). Further study is needed to identify the degree to which such ecosystem services scale with restoration size and age.

Our results highlight the need to increase measurement and reporting for data‐poor ecosystem services, including nutrient cycling, shoreline protection, water clarity, and dampened water flow, especially on restored and reference reefs. These services had low sample sizes, *p*‐values close to α = 0.05, or both, which suggests that their effects should be interpreted cautiously. Furthermore, despite the potential role of oyster reefs as carbon sinks (Fodrie et al., 2017), we found no studies that reported comparable measures of carbon storage on both restored reefs and either degraded or reference reefs. As oyster reefs are increasingly incorporated in global carbon accounting, more work is needed to compare sequestration potential on restored reefs to reference and degraded habitats.

### Implications for practice

Our meta‐analysis demonstrated that oyster restoration enhances a suite of ecosystem services relative to degraded habitats and approximates several natural restoration targets. Although we found that restored reefs matched reference reefs for many ecosystem services, restored systems rarely show complete recovery of ecological functions relative to natural systems (Benayas et al., 2009; Suding, 2011). Thus, conservation of existing natural reefs should still be prioritized even if restoration can approximate many of the ecosystem services provided by natural reefs (Jones et al., 2018). Furthermore, severe losses in the extent (64%) and biomass (88%) of historic oyster reefs in the United States mean that one must compare restored reefs to natural reference reefs whose population baselines have already shifted relative to historic levels, which are now likely unattainable with restoration (Lotze et al., 2011; Suding, 2011; zu Ermgassen, Spalding, Blake, et al., 2012). Likewise, overfishing and climate change have reduced the contemporary baseline for the abundance and size of many coastal fish and shellfish species and distorted expectations for pristine restoration targets (Dayton et al., 1998; Jackson et al., 2001; McClenachan, 2009).

Although we focused on the eastern oyster, oyster restoration projects outside of the United States are commonly modeled after successful restoration efforts with eastern oysters (Fitzsimons et al., 2019; zu Ermgassen et al., 2020). We expect that our findings will translate to other oyster species with similar life histories. As oyster restoration projects continue to expand internationally, it will be important to include control areas in restoration monitoring plans to support future synthesis of oyster restoration attributes at a global scale.

Despite the challenges associated with restoring a historically degraded system, the results of our meta‐analysis support the promise that restoring a valuable foundation species can substantially enhance a suite of ecosystem services relative to degraded systems and match those of restoration targets. More work is needed to identify the factors driving variation in the ecosystem services provided by restoration. Such knowledge can reveal why some projects do not meet restoration goals and help optimize future restoration to target specific ecosystem services.

## Supporting information

Appendix S1: Flow chart of publication search process following the Preferred Reporting Items for Systematic Reviews and Meta‐Analyses (PRISMA) standards for meta‐analysis reporting (Moher et al. 2009). Out of 1121 initially identified publications, we identified 106 publications that met our criteria for inclusion in the meta‐analysis.
Appendix S2: Checklist for the Preferred Reporting Items for Systematic Reviews and Meta‐Analyses (PRISMA) standards for meta‐analysis reporting (Moher et al. 2009).
Appendix S3: The number of screened publications that were excluded from the meta‐analysis and the reason for exclusion.
Appendix S4: List of meta‐data (authors, year, source, and title) for the 106 publications included in the meta‐analysis.
Appendix S5. Abundances of select taxa on restored reefs exceeded those on degraded reefs for orders of (A) fishes and (B) mollusks and worms, and families of (C) crabs and (D) shrimps. Points represent the mean log response ratio (LRR) for taxa abundance on restored reefs relative to degraded reefs. Positive LRRs indicate an increase in taxa abundance on restored reefs relative to paired degraded reefs. Error bars show 95% confidence intervals and asterisks denote effect sizes that differ from zero (p < 0.05). The number of papers and effect sizes (parenthetically) are shown next to each mean effect size. Dagger indicates effect sizes that are marginally different from zero (p < 0.1).
Appendix S6: Abundances of most taxa on restored reefs were similar to those on reference reefs for orders of (A) fishes and (B) mollusks, and families of (C) crabs and (D) shrimps. Points, error bars, asterisks, daggers and numbers as in Fig. S1.
Appendix S7: Environmental responses associated with habitat suitability, including dissolved oxygen, salinity, and temperature were similar between restored reefs and comparison reefs. Points, error bars, asterisks, daggers and numbers as in Fig. S1.
Appendix S8: Sensitivity analyses.
Appendix S9: Funnel plots of log response ratios (LRRs) for oyster production versus sample size indicated no signs of publication bias for LRRs calculated for (A) restored reefs relative to degraded reefs and (B) restored reefs relative to reference reefs.
Appendix S10: Funnel plots of log response ratios (LRRs) for habitat provisioning responses versus sample size indicated no signs of publication bias for LRRs calculated for restored reefs relative to degraded reefs and restored reefs relative to reference reefs for (A) community abundance, (B) community richness, (C) community abundance and (D) individual size (length). ‘I.D.’ indicates insufficient data for analysis (< 3 publications).
Appendix S11: Funnel plots of log response ratios (LRRs) for ecosystem functioning responses versus sample size indicated no signs of publication bias for LRRs calculated for restored reefs relative to degraded reefs and restored reefs relative to reference reefs for (A) nitrogen removal, (B) nutrients, (C) organic matter, (D) primary productivity, (E) water clarity, and (F) shoreline protection responses. ‘I. D.’ indicates insufficient data for analysis (< 2 publications).
Appendix S12: Caterpillar plots of mean log response ratio ± standard error for each publication for total oyster abundance on restored reefs relative to (A) degraded reefs and (B) reference reefs.
Appendix S13: Caterpillar plots of mean log response ratio ± standard error for each publication for community taxa abundance on restored reefs relative to (A) degraded reefs and (B) reference reefs.
Appendix S14: Caterpillar plots of mean log response ratio ± standard error for each publication for community taxa diversity on restored reefs relative to (A) degraded reefs and (B) reference reefs.
Appendix S15: Caterpillar plots of mean log response ratio ± standard error for each publication for nitrogen removal on restored reefs relative to degraded reefs.
Appendix S16: Caterpillar plots of mean log response ratio ± standard error for each publication for (A‐B) sediment nutrients and (C‐D) water column nutrients on restored reefs relative to degraded reefs and reference reefs.
Appendix S17: Caterpillar plots of mean log response ratio ± standard error for each publication for water clarity on restored reefs relative to (A) degraded reefs and (B) reference reefs.
Appendix S18: Caterpillar plots of mean log response ratio ± standard error for each publication for dampened hydrodynamics on restored reefs relative to (A) degraded reefs and (B) reference reefs and for (C) shoreline advance on restored reefs relative to degraded reefs.
Appendix S19: Oyster abundance on restored reefs relative to degraded and reference reefs for (A) variance‐weighted log response ratios, (B) sample size‐weighted log response ratios, and (C) Hedges’ *d*. Equally‐weighted means from the full dataset are reproduced in grey unfilled circles in (A) and (B) (from Figure 4, main text). Points represent mean values and error bars show 95% confidence intervals. The number of papers and effect sizes (parenthetically) are shown next to each mean effect size.
Appendix S20: Community abundance, richness, length and biomass on restored reefs relative to degraded and reference reefs for (A) variance‐weighted log response ratios, (B) sample size‐weighted log response ratios, and (C) Hedges’ *d*. Equally‐weighted means from the full dataset are reproduced in grey unfilled circles in (A) and (B) (from Figure 5, main text). Points, error bars, asterisks, and numbers as in Appendix S19. ‘I. D.’ indicates insufficient data for analysis (< 3 publications).
Appendix S21: Taxa group abundance for nekton, epifauna, infauna, birds, and plants on restored reefs relative to degraded and reference reefs for (A) variance‐weighted log response ratios, (B) sample size‐weighted log response ratios, and (C) Hedges’ *d*. Equally‐weighted means from the full dataset are reproduced in grey unfilled circles in (A) and (B) (from Figure 5, main text). Points, error bars, asterisks, and numbers as in Appendix S19. ‘I. D.’ indicates insufficient data for analysis (< 3 publications).
Appendix S22: (A) Nitrogen removal, (B) sediment and water column nutrients, (C) organic matter, and (D) chlorophyll concentrations, (E) water clarity, and (F) shoreline protection on restored reefs relative to degraded and reference reefs for sample size‐weighted log response ratios. Equally‐weighted means from the full dataset are reproduced in grey unfilled circles (from Figure 6, main text). Points, error bars, asterisks, and numbers as in Appendix S19. ‘I. D.’ indicates insufficient data for analysis (< 2 publications).
Appendix S23: (A) Nitrogen removal, (B) sediment and water column nutrients, (C) organic matter, and (D) chlorophyll concentrations, and (E) shoreline protection on restored reefs relative to degraded and reference reefs for Hedges’ *d*. Equally‐weighted means from the full dataset are reproduced in grey unfilled circles (from Figure 6, main text). Points, error bars, asterisks, and numbers as in Appendix S19. ‘I. D.’ indicates insufficient data for analysis (< 2 publications).
