# Supplementary material for: Meta‐analysis of ecosystem services associated with oyster restoration
Source: Conserv Biol. 2022 Sep 8;37(1):e13966. doi: 10.1111/cobi.13966 (PMC10087230; doi:10.1111/cobi.13966)
Supplement: Supplementary file 1 — Appendix S1: Flow chart of publication search process following the Preferred Reporting Items for Systematic Reviews and Meta‐Analyses (PRISMA) standards for meta‐analysis reporting (Moher et al. 2009). Out of 1121 initially identified publications, we identified 106 publications that met our criteria for inclusion in the meta‐analysis. Appendix S2: Checklist for the Preferred Reporting Items for Systematic Reviews and Meta‐Analyses (PRISMA) standards for meta‐analysis reporting (Moher et al. 2009). Appendix S3: The number of screened publications that were excluded from the meta‐analysis and the reason for exclusion. Appendix S4: List of meta‐data (authors, year, source, and title) for the 106 publications included in the meta‐analysis. Appendix S5. Abundances of select taxa on restored reefs exceeded those on degraded reefs for orders of (A) fishes and (B) mollusks and worms, and families of (C) crabs and (D) shrimps. Points represent the mean log response ratio (LRR) for taxa abundance on restored reefs relative to degraded reefs. Positive LRRs indicate an increase in taxa abundance on restored reefs relative to paired degraded reefs. Error bars show 95% confidence intervals and asterisks denote effect sizes that differ from zero (p < 0.05). The number of papers and effect sizes (parenthetically) are shown next to each mean effect size. Dagger indicates effect sizes that are marginally different from zero (p < 0.1). Appendix S6: Abundances of most taxa on restored reefs were similar to those on reference reefs for orders of (A) fishes and (B) mollusks, and families of (C) crabs and (D) shrimps. Points, error bars, asterisks, daggers and numbers as in Fig. S1. Appendix S7: Environmental responses associated with habitat suitability, including dissolved oxygen, salinity, and temperature were similar between restored reefs and comparison reefs. Points, error bars, asterisks, daggers and numbers as in Fig. S1. Appendix S8: Sensitivity analyses. Appendix S9: Funnel pl [file COBI-37-0-s001.docx]

Electronic Supplemental Material

**Meta-analysis of ecosystem services associated with oyster restoration**

Rachel S. Smith,^1^* Selina L. Cheng,^2^ Max C. N. Castorani^1^

^1^Department of Environmental Sciences, University of Virginia, Charlottesville, VA 22903.

*Correspondence to:

Rachel Smith

University of Virginia

Department of Environmental Sciences

291 McCormick Road

Charlottesville, VA 22903

[Rssmith218@gmail.com](mailto:Rssmith218@gmail.com)

**Appendix S1:** Flow chart of publication search process following the Preferred Reporting Items for Systematic Reviews and Meta-Analyses (PRISMA) standards for meta-analysis reporting (Moher et al. 2009). Out of 1121 initially identified publications, we identified 106 publications that met our criteria for inclusion in the meta-analysis.

Records identified through database searching
(n = 1,411)

## Screening

## Included

## Eligibility

## Identification

Additional records identified through other sources
(n = 290)

Records after duplicates removed
(n = 1,121)

Records screened
(n = 1,121)

Records excluded
(n = 865)

Full-text articles assessed for eligibility
(n = 256)

Full-text articles excluded
(n = 150)

Studies included in qualitative synthesis
(n = NA)

Studies included in quantitative synthesis (meta-analysis)
(n = 106)


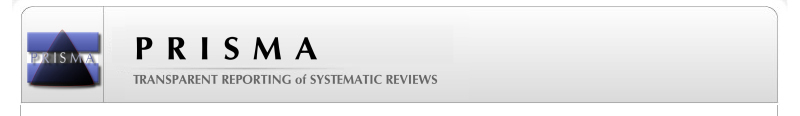


**PRISMA 2009 Flow Diagram**

**Appendix S2:** Checklist for the Preferred Reporting Items for Systematic Reviews and Meta-Analyses (PRISMA) standards for meta-analysis reporting (Moher et al. 2009).

| **Section/topic** | **#** | **Checklist item** | **Reported on page #** |
| --- | --- | --- | --- |
| **TITLE** | | |  |
| Title | 1 | Identify the report as a systematic review, meta-analysis, or both. | 1 |
| **ABSTRACT** | | |  |
| Structured summary | 2 | Provide a structured summary including, as applicable: background; objectives; data sources; study eligibility criteria, participants, and interventions; study appraisal and synthesis methods; results; limitations; conclusions and implications of key findings; systematic review registration number. | 1 |
| **INTRODUCTION** | | |  |
| Rationale | 3 | Describe the rationale for the review in the context of what is already known. | 2-4 |
| Objectives | 4 | Provide an explicit statement of questions being addressed with reference to participants, interventions, comparisons, outcomes, and study design (PICOS). | 4 |
| **METHODS** | | |  |
| Protocol and registration | 5 | Indicate if a review protocol exists, if and where it can be accessed (e.g., Web address), and, if available, provide registration information including registration number. | 6 |
| Eligibility criteria | 6 | Specify study characteristics (e.g., PICOS, length of follow-up) and report characteristics (e.g., years considered, language, publication status) used as criteria for eligibility, giving rationale. | 6-7 |
| Information sources | 7 | Describe all information sources (e.g., databases with dates of coverage, contact with study authors to identify additional studies) in the search and date last searched. | 6-7 |
| Search | 8 | Present full electronic search strategy for at least one database, including any limits used, such that it could be repeated. | 7 |
| Study selection | 9 | State the process for selecting studies (i.e., screening, eligibility, included in systematic review, and, if applicable, included in the meta-analysis). | 6-7 |
| Data collection process | 10 | Describe method of data extraction from reports (e.g., piloted forms, independently, in duplicate) and any processes for obtaining and confirming data from investigators. | 7-8 |
| Data items | 11 | List and define all variables for which data were sought (e.g., PICOS, funding sources) and any assumptions and simplifications made. | 5-9 |
| Risk of bias in individual studies | 12 | Describe methods used for assessing risk of bias of individual studies (including specification of whether this was done at the study or outcome level), and how this information is to be used in any data synthesis. | 9 |
| Summary measures | 13 | State the principal summary measures (e.g., risk ratio, difference in means). | 8-9 |
| Synthesis of results | 14 | Describe the methods of handling data and combining results of studies, if done, including measures of consistency (e.g., I^2^) for each meta-analysis. | 8-9 |

| **Section/topic** | **#** | **Checklist item** | **Reported on page #** |
| --- | --- | --- | --- |
| Risk of bias across studies | 15 | Specify any assessment of risk of bias that may affect the cumulative evidence (e.g., publication bias, selective reporting within studies). | 9 |
| Additional analyses | 16 | Describe methods of additional analyses (e.g., sensitivity or subgroup analyses, meta-regression), if done, indicating which were pre-specified. | 9 |
| **RESULTS** | | |  |
| Study selection | 17 | Give numbers of studies screened, assessed for eligibility, and included in the review, with reasons for exclusions at each stage, ideally with a flow diagram. | 10 |
| Study characteristics | 18 | For each study, present characteristics for which data were extracted (e.g., study size, PICOS, follow-up period) and provide the citations. | Table S3 |
| Risk of bias within studies | 19 | Present data on risk of bias of each study and, if available, any outcome level assessment (see item 12). | Appendix S8-11;19-23 |
| Results of individual studies | 20 | For all outcomes considered (benefits or harms), present, for each study: (a) simple summary data for each intervention group (b) effect estimates and confidence intervals, ideally with a forest plot. | Appendix S12-18 |
| Synthesis of results | 21 | Present results of each meta-analysis done, including confidence intervals and measures of consistency. | 10-14 |
| Risk of bias across studies | 22 | Present results of any assessment of risk of bias across studies (see Item 15). | 11, Appendix S8-11;19-23 |
| Additional analysis | 23 | Give results of additional analyses, if done (e.g., sensitivity or subgroup analyses, meta-regression [see Item 16]). | 11, Appendix S8-11;19-23 |
| **DISCUSSION** | | |  |
| Summary of evidence | 24 | Summarize the main findings including the strength of evidence for each main outcome; consider their relevance to key groups (e.g., healthcare providers, users, and policy makers). | 14-19 |
| Limitations | 25 | Discuss limitations at study and outcome level (e.g., risk of bias), and at review-level (e.g., incomplete retrieval of identified research, reporting bias). | 14-19 |
| Conclusions | 26 | Provide a general interpretation of the results in the context of other evidence, and implications for future research. | 19-20 |
| **FUNDING** | | |  |
| Funding | 27 | Describe sources of funding for the systematic review and other support (e.g., supply of data); role of funders for the systematic review. | Cover letter |

**Appendix S3:** The number of screened publications that were excluded from the meta-analysis and the reason for exclusion.

| **Reasons for exclusion** | **Number of publications excluded** |
| --- | --- |
| No comparison reef | 54 |
| No restored reef | 311 |
| No target response variable | 14 |
| Not field study | 325 |
| Reported on non-target taxa | 287 |
| Paper not accessible | 24 |

**Appendix S4:** List of meta-data (authors, year, source, and title) for the 106 publications included in the meta-analysis.

|  | **Authors** | **Year** | **Journal** | **Title** |
| --- | --- | --- | --- | --- |
| 1 | Anderson *et al.* | 2019 | Restoration Ecology | Oyster reef enhancement utilizing gardened oysters in a subtropical estuary |
| 2 | Arnaldi *et al.* | 2018 | Restoration Ecology | Effects of time and harvest on genetic diversity of natural and restored oyster reefs |
| 3 | Arve J. | 1960 | Chesapeake Bay | Preliminary report on attracting fish by oyster-shell plantings in Chincoteague Bay, Maryland |
| 4 | Birch, A.P & Walters, L. | 2012 | Gray literature | Restoring intertidal oyster reefs in Mosquito Lagoon: the evolution of a successful model |
| 5 | Blomberg *et al.* | 2018 | Ecological Engineering | Habitat assessment of a restored oyster reef in South Texas |
| 6 | Booth, D.M. & Heck, K.L. | 2009 | Marine Ecology Progress Series | Effects of the American oyster *Crassostrea virginica* on growth rates of the seagrass *Halodule wrightii* |
| 7 | Brown, L.A. | 2012 | Graduate thesis | Oyster reef restoration in the northern Gulf of Mexico: effect of substrate and age on ecosystem services |
| 8 | Brown *et al.* | 2014 | Restoration Ecology | Oyster reef restoration in the northern Gulf of Mexico: effect of artificial substrate and age on nekton and benthic macroinvertebrate assemblage use |
| 9 | Brumbaugh *et al.* | 2000 | Journal of Shellfish Research | Making a case for community-based oyster restoration: An example from Hampton Roads, Virginia, USA |
| 10 | Chambers *et al.* | 2018 | Estuaries and Coasts | How well do restored intertidal oyster reefs support key biogeochemical properties in a coastal lagoon? |
| 11 | Clarke *et al.* | 1999 | Symposium proceeedings | Dredged material as a substrate for fisheries habitat establishment in coastal waters |
| 12 | Coen *et al.* | 1999 | Symposium proceedings | Intertidal oyster reef studies in South Carolina: design, sampling, and experimental focus for evaluating habitat value and function |
| 13 | Coen L.D. & Luckenbach M.W. | 2000 | Ecological Engineering | Developing success criteria and goals for evaluating oyster reef restoration: ecological function or resource exploitation? |
| 14 | Colden *et al.* | 2016 | Estuaries and Coasts | Sediment suspension and deposition across restored oyster reefs of varying orientation to flow: implications for restoration |
| 15 | De Santiago *et al.* | 2019 | Restoration Ecology | Rapid development of a restored oyster reef facilitates habitat provision for estuarine fauna |
| 16 | Dillon *et al.* | 2015 | Marine Ecology Progress Series | Functional equivalence of constructed and natural intertidal eastern oyster reef habitats in a northern Gulf of Mexico estuary |
| 17 | Drexler *et al.* | 2014 | Estuaries and Coasts | Biological assessment of Eastern Oysters (*Crassostrea virginica*) inhabiting reef, mangrove, seawall, and restoration substrates |
| 18 | Dunnigan, S. K. | 2015 | Graduate thesis | Habitat value of restored intertidal shoreline for fish and macrobenthic communities in Northeast Florida |
| 19 | Finelli *et al.* | 2013 | Grey literature | To seed or not to seed: the value of seeding restored oyster reefs for ecosystem function |
| 20 | Frederick P. *et al.* | 2016 | Journal of Shellfish Research | Reversing a rapid decline in oyster reefs: effects of durable substrate on oyster populations, elevations, and aquatic bird community composition |
| 21 | Gedan K.B. *et al.* | 2014 | Restoration Ecology | Accounting for multiple foundation species in oyster reef restoration benefits |
| 22 | George L.M. *et al.* | 2015 | Journal of Coastal Conservation | Oyster reef restoration: effect of alternative substrates on oyster recruitment and nekton habitat use |

|  | **Authors** | **Year** | **Journal** | **Title** |
| --- | --- | --- | --- | --- |
| 23 | Geraldi N.R. *et al.* | 2009 | Marine Ecology Progress Series | Can habitat restoration be redundant? Response of mobile fishes and crustaceans to oyster reef restoration in marsh tidal creeks |
| 24 | Grabowski J.H. *et al.* | 2005 | Ecology | How habitat setting influences restored oyster reef communities |
| 25 | Gregalis K.C. *et al.* | 2009 | Transactions of the American Fisheries Society | Restored oyster reef location and design affect responses of resident and transient fish, crab, and shellfish species in Mobile Bay, Alabama |
| 26 | Grizzle R. *et al.* | 2008 | Estuaries and Coasts | Seston removal by natural and constructed intertidal Eastern Oyster (*Crassostrea virginica)* reefs: a comparison with previous laboratory studies, and the value of in situ methods |
| 27 | Hanke M.H *et al.* | 2015 | Journal of Shellfish Research | Oyster utilization and host variation of the oyster pea crab (*Zaops ostreum*) |
| 28 | Hanke M.H *et al.* | 2017 | Marine Ecology Progress Series | The influence of habitat characteristics on intertidal oyster *Crassostrea virginica* populations |
| 29 | Hanke M.H *et al.* | 2017 | Marine Ecology Progress Series | The effects of intertidal oyster reef habitat characteristics on faunal utilization |
| 30 | Harding J.M. & Mann R. | 1999 | Bulletin of Marine Science | Fish species richness in relation to restored oyster reefs, Piankatank River, Virginia |
| 31 | Harding J.M. & Mann R. | 2001 | Environmental Biology of Fishes | Diet and habitat use by bluefish, *Pomatomus saltatrix*, in a Chesapeake Bay estuary |
| 32 | Harding J.M. & Mann R. | 2001 | Journal of Shellfish Research | Oyster reefs as fish habitat: opportunistic use of restored reefs by transient fishes |
| 33 | Harding J.M. & Mann R. | 2003 | Bulletin of Marine Science | Influence of habitat on diet and distribution of striped bass (*Morone saxatilis*) in a temperate estuary |
| 34 | Harding J.M. & Mann R. | 2010 | Bulletin of Marine Science | Observations of distribution, size, and sex ratio of mature blue crabs, *Callinectes sapidus*, from a Chesapeake Bay tributary in relation to oyster habitat and environmental factors |
| 35 | Harding J.M. *et al.* | 2012 | Northeastern Naturalist | Comparison of *Crassostrea virginica* Gmelin (Eastern Oyster) recruitment on constructed reefs and adjacent natural oyster bars over decadal time scales |
| 36 | Harris K. | 2019 | Graduate thesis | Oyster reef restoration: impacts on infaunal communities in a shallow water estuary |
| 37 | Harwell H.D. *et al.* | 2011 | Journal of Experimental Marine Biology and Ecology | Landscape aspects of oyster reefs: effects of fragmentation on habitat utilization |
| 38 | Hassett M.C. | 2015 | Graduate thesis | The influence of Eastern Oyster (*Crassostrea virginica*) reef restoration on nitrogen cycling in a eutrophic estuary |
| 39 | Hoellein T.J. & Zarnoch C.B | 2014 | Ecological Applications | Effect of eastern oysters (*Crassostrea virginica*) on sediment carbon and nitrogen dynamics in an urban estuary |
| 40 | Hubbard A.B. *et al.* | 2015 | Marine Ecology Progress Series | Effects of larval swimming behavior on the dispersal and settlement of the eastern oyster *Crassostrea virginica* |
| 41 | Humphries A.T. *et al.* | 2016 | Frontiers in Marine Science | Directly measured denitrification reveals oyster aquaculture and restored oyster reefs remove nitrogen at comparable high rates |
| 42 | Humphries A.T. *et al.* | 2015 | PeerJ | Oyster reef restoration supports increased nekton biomass and potential commercial fishery value |
| 43 | Humphries A.T. *et al.* | 2011 | Journal of Experimental Marine Biology and Ecology | Testing the effect of habitat structure and complexity on nekton assemblages using experimental oyster reefs |

|  | **Authors** | **Year** | **Journal** | **Title** |
| --- | --- | --- | --- | --- |
| 44 | Jaris H. *et al.* | 2019 | Conservation Genetics | Assessing the contribution of aquaculture and restoration to wild oyster populations in a Rhode Island coastal lagoon |
| 45 | Jud Z.R. & Layman C.A. | 2020 | Food Webs | Changes in motile benthic faunal community structure following large-scale oyster reef restoration in a subtropical estuary |
| 46 | Karp M.A. *et al.* | 2018 | Marine Ecology Progress Series | Faunal communities on restored oyster reefs: effects of habitat complexity and environmental conditions |
| 47 | Keller D.A. *et al.* | 2019 | Restoration Ecology | Salt marsh shoreline geomorphology influences the success of restored oyster reefs and use by associated fauna |
| 48 | Kellogg M.L. *et al* | 2016 | Gray literature | Integrated assessment of oyster reef ecosystem services: fish and crustacean utilization and trophic linkages |
| 49 | Kellogg M.L. *et al* | 2013 | Marine Ecology Progress Series | Denitrification and nutrient assimilation on a restored oyster reef |
| 50 | Kenworthy, M.D. | 2019 | Graduate thesis | The movement ecology of large, mobile fishes in North Carolina estuaries |
| 51 | Kingsley-Smith, P.R. *et al.* | 2012 | Journal of Shellfish Research | Habitat use of intertidal Eastern Oyster (*Crassostrea virginica*) reefs by nekton in South Carolina estuaries |
| 52 | La Peyre, M.K. *et al.* | 2014 | Ecological Engineering | Temporal variation in development of ecosystem services from oyster reef restoration |
| 53 | La Peyre, M.K. *et al.* | 2013 | Grey literature | Preliminary assessment of bioengineered fringing shoreline reefs in Grand Isle and Breton Sound, Louisiana |
| 54 | La Peyre, M.K. *et al.* | 2013 | Grey literature | Baseline data for evaluating the development trajectory and provision of ecosystem services by created fringing oyster reefs in Vermilion Bay, Louisiana |
| 55 | La Peyre, M.K. *et al._­_* | 2015 | PeerJ | Assessing shoreline exposure and oyster habitat suitability maximizes potential success for sustainable shoreline protection using restored oyster reefs |
| 56 | La Peyre, M.K. *et al.* | 2014 | Ocean and Coastal Management | Oyster reef restoration in the northern Gulf of Mexico: extent, methods and outcomes |
| 57 | Layman C.A. *et al.* | 2009 | Gray literature | Loxahatchee River oyster restoration & monitoring project |
| 58 | Lehnert R.L. & Allen D.M. | 2002 | Estuaries | Nekton use of subtidal oyster shell habitat in a southeastern US estuary |
| 59 | Lenihan H.S. *et al.* | 2001 | Ecological Applications | Cascading of habitat degradation: oyster reefs invaded by refugee fishes escaping stress |
| 60 | Locher, B.J. | 2019 | Graduate thesis | Understanding sediment biogeochemistry and the role of juvenile oysters on recently restored Eastern Oyster reefs |
| 61 | Locher, B.J *et al.* | 2020 | Estuaries and Coasts | Juvenile oyster (*Crassostrea virginica*) biodeposits contribute to a rapid rise in sediment nutrients on restored intertidal oyster reefs (Mosquito Lagoon, FL, USA) |
| 62 | Luckenbach M.W. *et al.* | 2005 | Journal of Coastal Research | Oyster reef habitat restoration: relationships between oyster abundance and community development based on two studies in Virginia and South Carolina |
| 63 | Marshall D.A. *et al.* | 2019 | Estuarine, Coastal, and Shelf Science | Salinity disturbance affects faunal community composition and organic matter on a restored *Crassostrea virginica* oyster reef |
| 64 | Meyer D.L. *et al.* | 2000 | Estuaries | Faunal utilization of created intertidal Eastern oyster (*Crassostrea virginica*) reefs in the souteastern United States |
| 65 | Milbrandt E.C. *et al.* | 2015 | Ecological Engineering | A multiple habitat restoration strategy in a semi-enclosed Florida embayment, combining hydrologic restoration, mangrove propagule plantings and oyster substrate additions |

|  | **Authors** | **Year** | **Journal** | **Title** |
| --- | --- | --- | --- | --- |
| 66 | Moksnes P. & Heck K.L. | 2006 | Marine Ecology Progress Series | Relative importance of habitat selection and predation for the distribution of blue crab megalopae and young juveniles |
| 67 | Nelson K.A. *et al.* | 2004 | Journal of Experimental Marine Biology and Ecology | Using transplanted oyster (*Crassostrea virginica*) beds to improve water quality in small tidal creeks: a pilot study |
| 68 | Nestlerode J.A. | 2004 | Graduate thesis | Evaluating restored oyster reefs in Chesapeake Bay: How habitat structure influences ecological function |
| 69 | O'Beirn F.X. *et al.* | 1999 | Gray literature | Ecological functions of constructed oyster reefs along an environmental gradient in Chesapeake Bay: Final report |
| 70 | Onorevole K.M. *et al.* | 2018 | Ecological Engineering | Living shorelines enhance nitrogen removal capacity over time |
| 71 | Peters, J.W. *et al.* | 2017 | Frontiers in Marine Science | Oyster demographics in harvested reefs vs. no-take reserves: implications for larval spillover and restoration success |
| 72 | Pfirrmann B.W. *et al.* | 2019 | Marine Ecology Progress Series | Ecosystem services of restored oyster reefs in a Chesapeake Bay tributary: abundance and foraging of estuarine fishes |
| 73 | Piazza B.P. *et al.* | 2005 | Restoration Ecology | The potential for created oyster shell reefs as a sustainable shoreline protection strategy in Louisiana |
| 74 | Pierson K.P *et al.* | 2014 | Transactions of the American Fisheries Society | Response of estuarine fish to large-scale oyster reef restoration |
| 75 | Plunket J. *et al.* | 2005 | Bulletin of Marine Science | Oyster beds as fish and macroinvertebrate habitat in Barataria Bay, Louisiana |
| 76 | Plutchak R. *et al.* | 2010 | Estuaries and Coasts | Impacts of oyster reef restoration on primary productivity and nutrient dynamics in tidal creeks of the North Central Gulf of Mexico |
| 77 | Powers S.P. *et al.* | 2009 | Marine Ecology Progress Series | Success of constructed oyster reefs in no-harvest sanctuaries: implications for restoration |
| 78 | Ravit, B. *et al.* | 2012 | Environmental Practice | Eastern oysters (*Crassostrea virginica*) in the Hudson-Raritan estuary: restoration research and shellfishery policy |
| 79 | Rezek R.J. *et al.* | 2017 | Marine Biology | How does a restored oyster reef develop? An assessment based on stable isotopes and community metrics |
| 80 | Ricci S.W. *et al.* | 2017 | Plos One | Oyster toadfish (*Opsanus tau*) boatwhistle call detection and patterns within a large-scale oyster restoration site |
| 81 | Ridge J.T. *et al.* | 2017 | Ecology and Evolution | Evidence of exceptional oyster-reef resilience to fluctuations in sea level |
| 82 | Rodney W.S. *et al.* | 2006 | Journal of Experimental Marine Biology and Ecology | Comparisons of macrofaunal assemblages on restored and non-restored oyster reefs in mesohaline regions of Chesapeake Bay in Maryland |
| 83 | Rutledge, K.M. *et al.* | 2018 | Estuaries and Coasts | Fish utilization of created vs. natural oyster reefs (*Crassostrea virginica*) |
| 84 | Schulte D.M. *et al.* | 2009 | Science | Unprecedented restoration of a native oyster metapopulation |
| 85 | Scyphers S.B. *et al.* | 2015 | Environmental Management | Ecological value of submerged breakwaters for habitat enhancement on a residential scale |
| 86 | Scyphers S.B. *et al.* | 2011 | Plos One | Oyster reefs as natural breakwaters mitigate shoreline loss and facilitate fisheries |
| 87 | Searles A. | 2019 | Graduate thesis | Assessing the impact of oyster reef and living shoreline restoration on macroinvertebrate community assemblages in Mosquito Lagoon, Florida |

|  | **Authors** | **Year** | **Journal** | **Title** |
| --- | --- | --- | --- | --- |
| 88 | Shaffer M. *et al.* | 2019 | Florida Field Naturalist | Does intertidal oyster reef restoration affect avian community structure and behavior in a shallow estuarine system? A post-restoration analysis |
| 89 | Sharma S. *et al.* | 2016 | Restoration Ecology | Do restored oyster reefs benefit seagrasses? An experimental study in the northern Gulf of Mexico |
| 90 | Simonsen K.A. *et al.* | 2013 | Bulletin of Marine Science | Effects of an inshore artificial reef on the trophic dynamics of three species of estuarine fish |
| 91 | Smyth A.R. *et al.* | 2016 | Marine Ecology Progress Series | Biological activity exceeds biogenic structure in influencing sediment nitrogen cycling in experimental oyster reefs |
| 92 | Smyth A.R. *et al.* | 2018 | Estuaries and Coasts | Differential effects of bivalves on sediment nitrogen cycling in a shallow coastal bay |
| 93 | Smyth A.R. *et al.* | 2015 | Journal of Applied Ecology | Habitat context influences nitrogen removal by restored oyster reefs |
| 94 | Sticklin, A.G. *et al.* | 2010 | Gulf and Caribbean Research | Do small, patchy, constructed intertidal oyster reefs reduce salt marsh erosion as well as natural reefs? |
| 95 | Stunz G.W. *et al.* | 2010 | Marine Ecology Progress Series | Relative value of oyster reef as habitat for estuarine nekton in Galveston Bay, Texas |
| 96 | Taubenheim TA | 2015 | Graduate thesis | Evaluation of biological and physical parameters of natural and restored oyster reefs in Georgia |
| 97 | Taylor, J.C. | 2008 | Graduate thesis | Evaluation of the ecological value of constructed intertidal oyster reefs and aquaculture structures in Delaware Bay: habitat utilization by motile macrofauna |
| 98 | Taylor, J.C. & Bushek D. | 2008 | Marine Ecology Progress Series | Intertidal oyster reefs can persist and function in a temperate North American Atlantic estuary |
| 99 | Tolley, G.S.*et al.* | 2005 | Journal of Shellfish Research | The role of oysters in habitat use of oyster reefs by resident fishes and decapod crustaceans |
| 100 | Troast, B.V. | 2019 | Graduate thesis | Exploring multi-scale variation of fish community diversity in a dynamic coastal estuary |
| 101 | van Montrans J.*et al.* | 2003 | Marine Ecology Progress Series | Substrate selection by blue crab *Callinectes sapidus* megalopae and first juvenile instars |
| 102 | Volaric M.P. *et al.* | 2018 | Marine Ecology Progress Series | Oxygen metabolism of intertidal oyster reefs measured by aquatic eddy covariance |
| 103 | Wenner E. *et al.* | 1996 | Journal of Shellfish Research | A method for quantitatively sampling nekton on intertidal oyster reefs |
| 104 | Whitman E.R. *et al.* | 2012 | Marine Ecology Progress Series | Benthic flow environments affect recruitment of *Crassostrea virginica* larvae to an intertidal oyster reef |
| 105 | Wiberg P.L. *et al.* | 2019 | Estuaries and Coasts | Wave attenuation by oyster reefs in shallow coastal bays |
| 106 | Ziegler S.L. *et al.* | 2018 | Restoration Ecology | Effects of landscape setting on oyster reef structure and function largely persist more than a decade post-restoration |

**Appendix S5.** Abundances of select taxa on restored reefs exceeded those on degraded reefs for orders of (A) fishes and (B) mollusks and worms, and families of (C) crabs and (D) shrimps. Points represent the mean log response ratio (LRR) for taxa abundance on restored reefs relative to degraded reefs. Positive LRRs indicate an increase in taxa abundance on restored reefs relative to paired degraded reefs. Error bars show 95% confidence intervals and asterisks denote effect sizes that differ from zero (p < 0.05). The number of papers and effect sizes (parenthetically) are shown next to each mean effect size. Dagger indicates effect sizes that are marginally different from zero (p < 0.1).
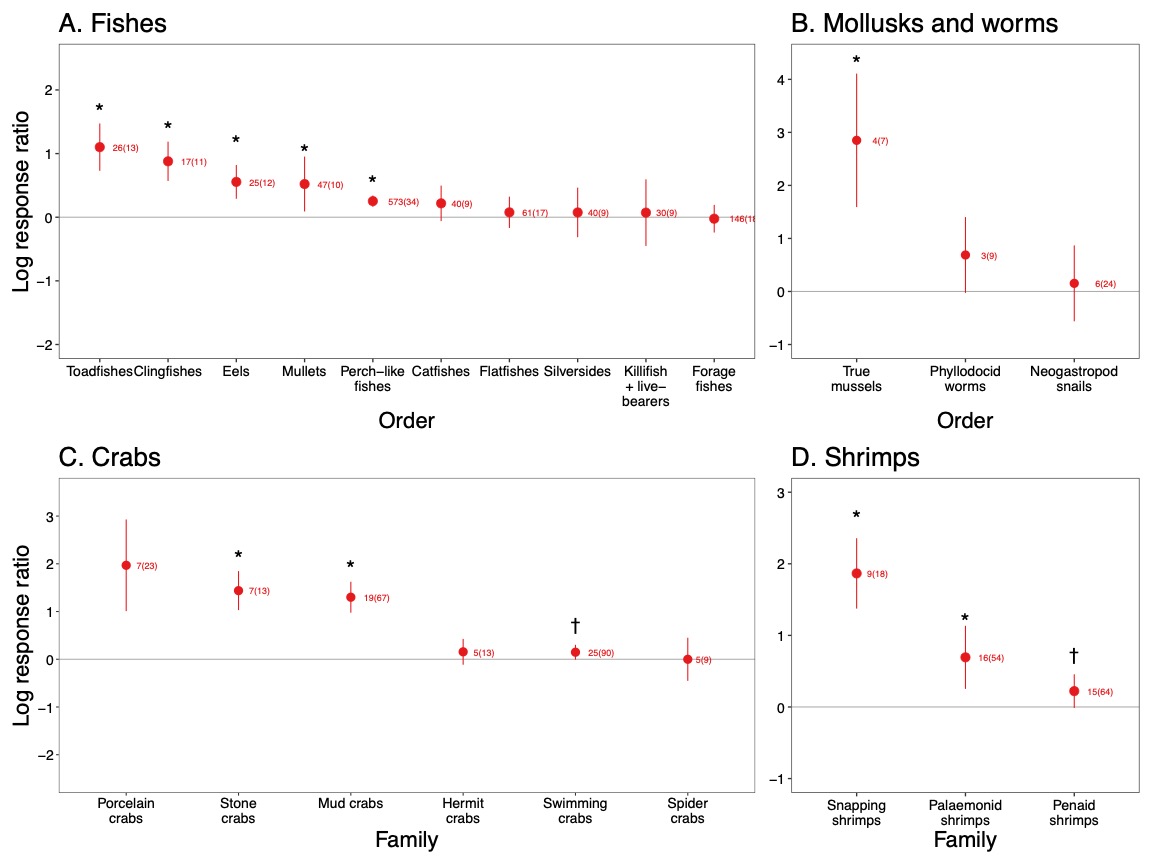


**Appendix S6:** Abundances of most taxa on restored reefs were similar to those on reference reefs for orders of (A) fishes and (B) mollusks, and families of (C) crabs and (D) shrimps. Points, error bars, asterisks, daggers and numbers as in Fig. S1.


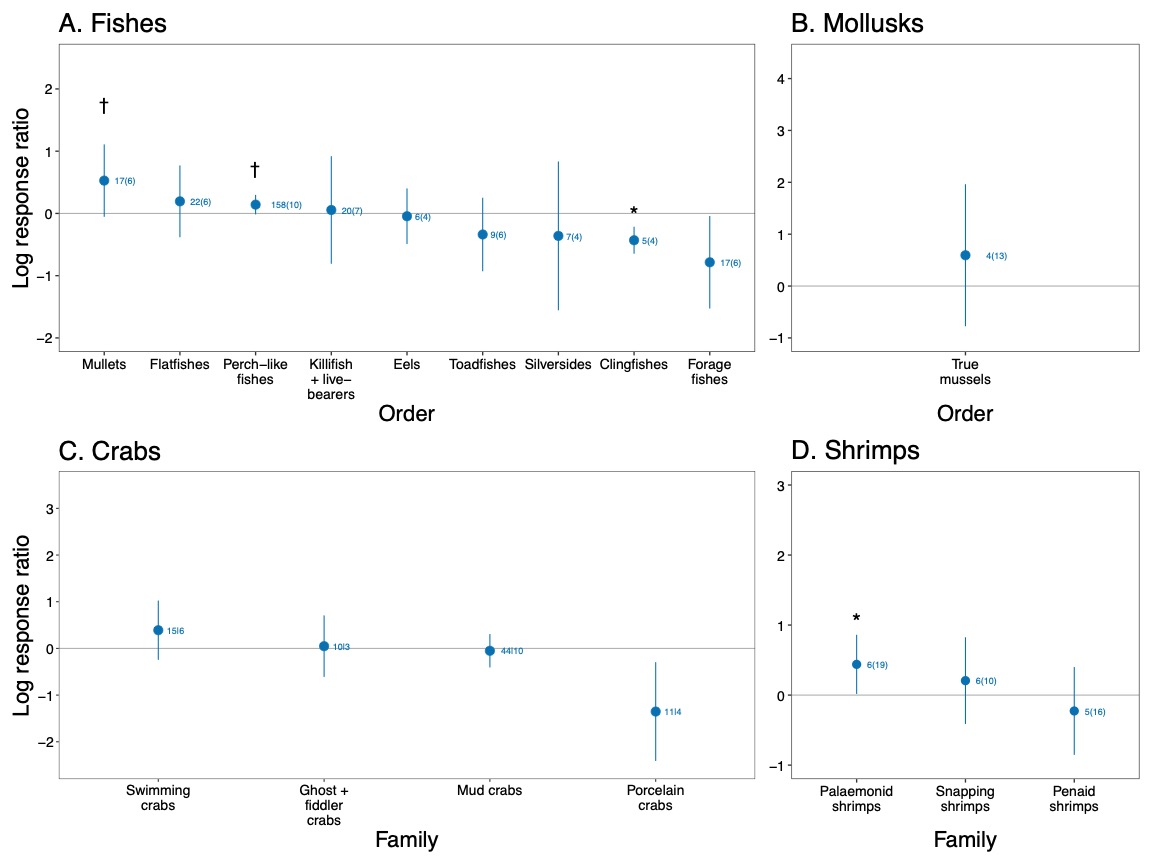


**Appendix S7**: Environmental responses associated with habitat suitability, including dissolved oxygen, salinity, and temperature were similar between restored reefs and comparison reefs. Points, error bars, asterisks, daggers and numbers as in Fig. S1.


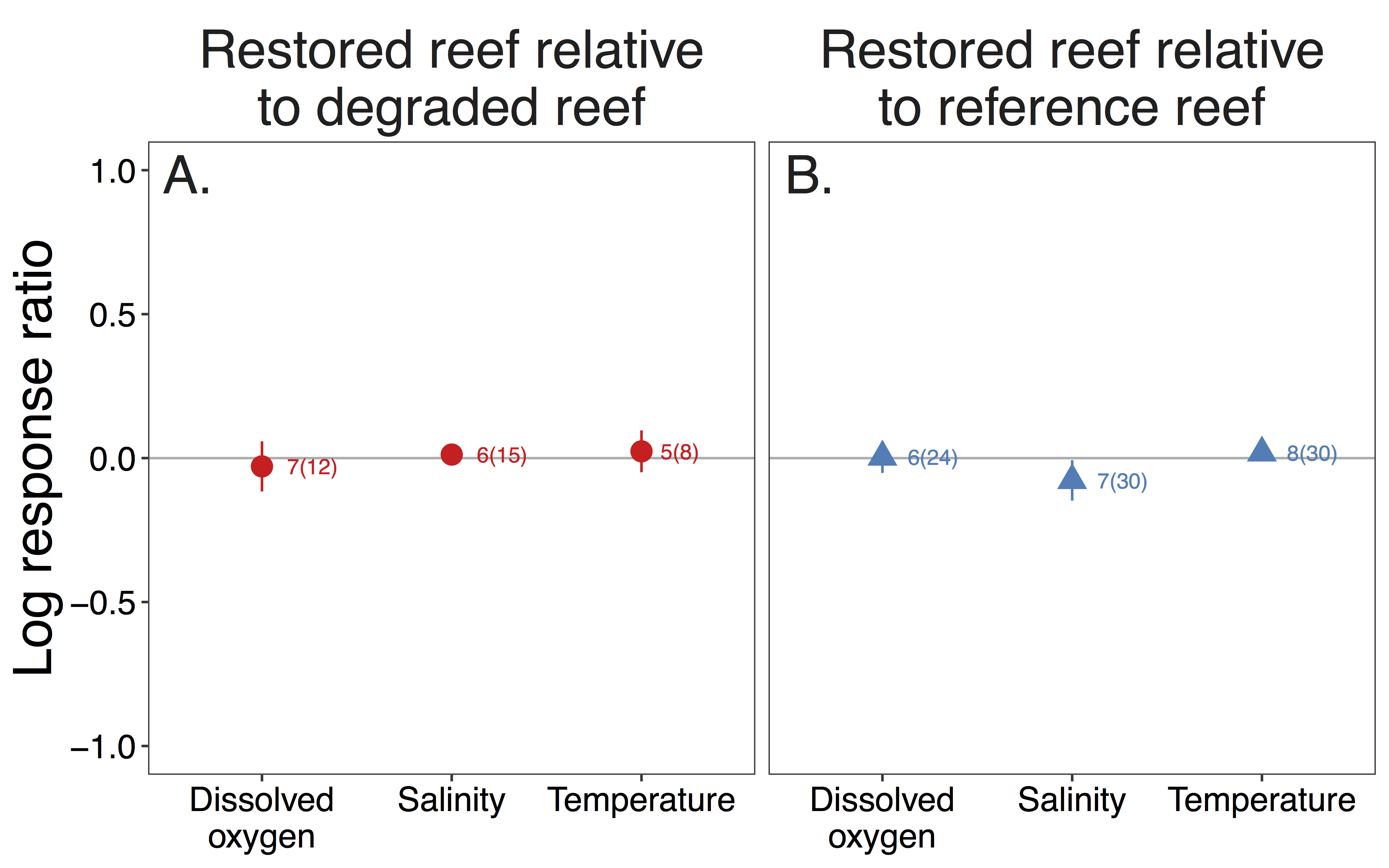


**Appendix S8: Sensitivity analyses**

**Supporting methods:**

*Effect size metrics:*

We chose to calculate log response ratios (LRRs) as the primary effect size metric because measures of variation were not available or estimable for many of the ecological responses. However, to assess the robustness of this metric, we also calculated complementary effect sizes that incorporate variance, including Hedges’ *d* (Hedges and Olkin 1984) and LRRs weighted by the inverse of the variance and sample size.

We calculated Hedge’s *d* as:

| 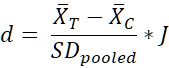 | (1) |
| --- | --- |

where is the mean response value from restored reefs, is the mean response value from comparison reefs (degraded or reference) and is the pooled standard deviation (based on the individual standard deviations of the two groups). *J* is the correction for small sample size:


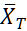

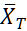

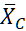

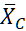

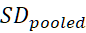

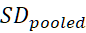


| 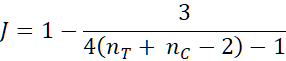 | (2) |
| --- | --- |

We calculated the variance of *d* as:

| 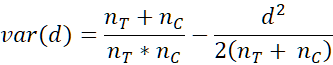 | (3) |
| --- | --- |

Because the Hedge’s *d* calculation includes variance measures (i.e., standard deviation), we could only calculate *d* for a subset of the data (*n* = 1746 effect sizes for Hedges’ *d* vs. 4093 effect sizes in the main analysis). Similarly, calculations of LRRs weighted by the inverse of the variance and the sample size used a subset of the data (variance-weighted: *n =* 2592 effect sizes; sample-weighted: *n =* 4051 effect sizes).

*Tests of bias and sensitivity:*

We used funnel plots to assess the potential for publication bias and found no evidence of asymmetry. We also used a ‘drop-one’ approach to assess whether any study exerted undue influence on the overall mean LRR by systematically dropping each study, re-calculating the LRR for that response, and then comparing the new and old mean LRR values with a two-sided *t-*test (as in Lefcheck et al. 2019). In only two cases did removing a study significantly change the mean *LRR* (p < 0.05), but the resulting means were both still in the same direction (positive).

Lastly, we calculated the ‘fail-safe N’ to determine how many non-significant, unpublished studies (LRR = 0) would be needed to remove a significant overall effect size (Rosenthal 1979). We used a threshold fail-safe number of 5*N* + 10 to determine the potential presence of publication bias (Rosenthal 1979). Across all studies, on average 624,297 non-significant studies would need to be added to the meta-analysis to change results from statistical significance to non-significance, exceeding the threshold number of 540 studies. We also calculated separate fail-safe numbers for papers reporting responses for restored reefs relative to degraded reefs and restored reefs relative to reference reefs. For studies that measured responses on restored and degraded reefs, on average 545,262 non-significant studies would be needed, exceeding the threshold number of 405 studies. For studies that measured responses on restored and reference reefs, on average 1537 non-significant studies would be needed, exceeding the threshold number of 215 studies. Together, these tests indicate that our results are robust to publication bias.

**Appendix S9:** Funnel plots of log response ratios (LRRs) for oyster production versus sample size indicated no signs of publication bias for LRRs calculated for (A) restored reefs relative to degraded reefs and (B) restored reefs relative to reference reefs.

**Appendix S10:** Funnel plots of log response ratios (LRRs) for habitat provisioning responses versus sample size indicated no signs of publication bias for LRRs calculated for restored reefs relative to degraded reefs and restored reefs relative to reference reefs for (A) community abundance, (B) community richness, (C) community abundance and (D) individual size (length). ‘I.D.’ indicates insufficient data for analysis (< 3 publications).


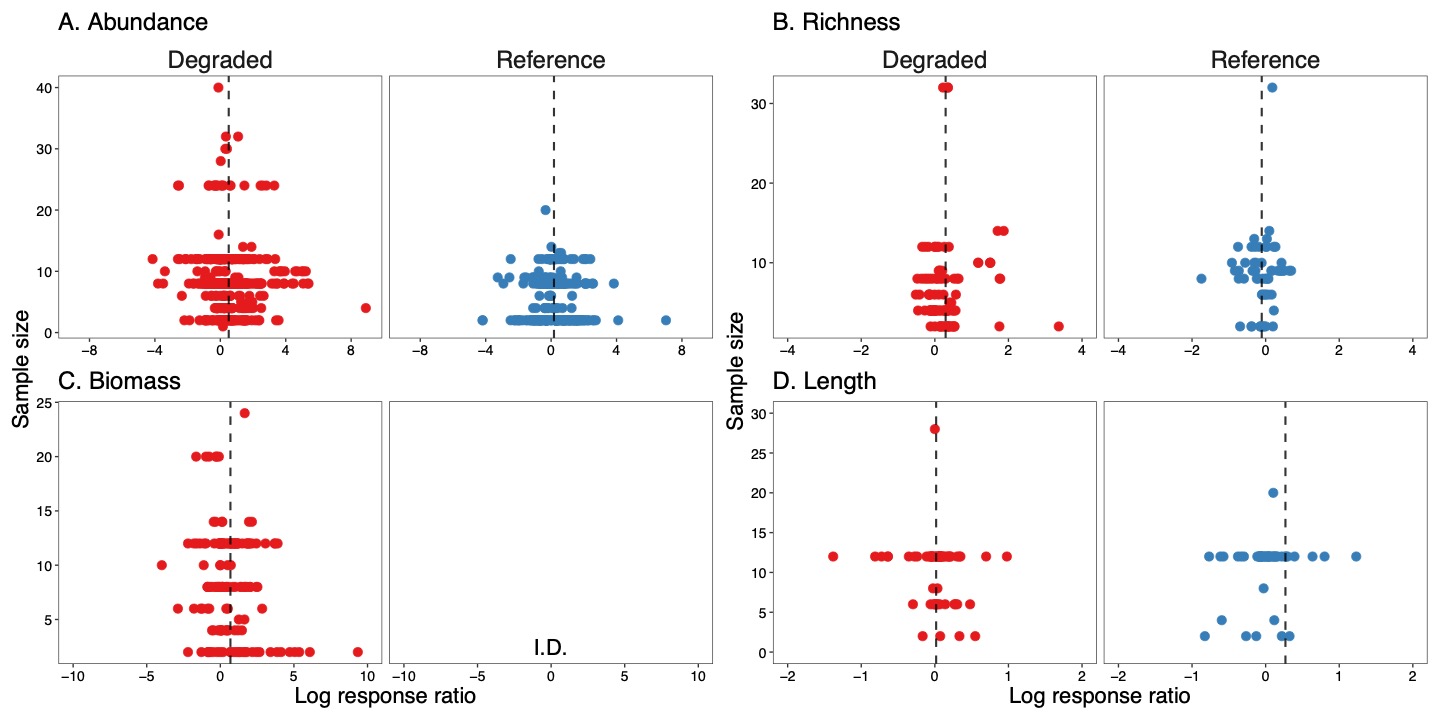


**Appendix S11:** Funnel plots of log response ratios (LRRs) for ecosystem functioning responses versus sample size indicated no signs of publication bias for LRRs calculated for restored reefs relative to degraded reefs and restored reefs relative to reference reefs for (A) nitrogen removal, (B) nutrients, (C) organic matter, (D) primary productivity, (E) water clarity, and (F) shoreline protection responses. ‘I. D.’ indicates insufficient data for analysis (< 2 publications).

**
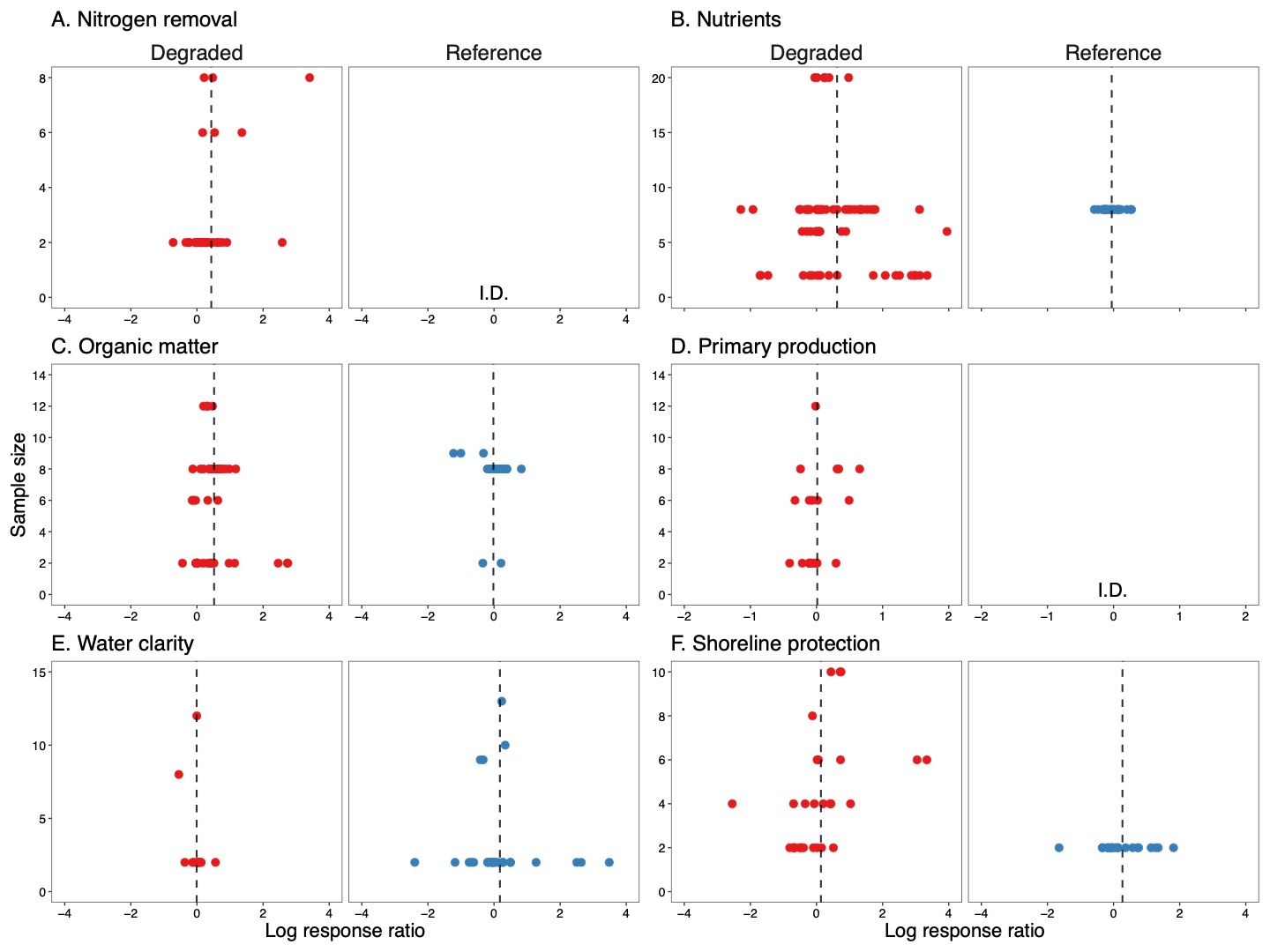
**

**Appendix S12:** Caterpillar plots of mean log response ratio ± standard error for each publication for total oyster abundance on restored reefs relative to (A) degraded reefs and (B) reference reefs.

**
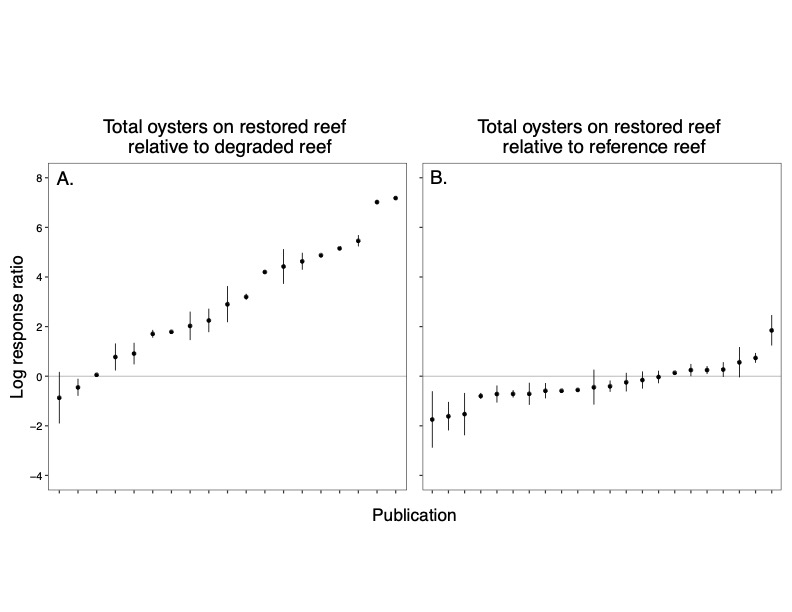
**

**Appendix S13:** Caterpillar plots of mean log response ratio ± standard error for each publication for community taxa abundance on restored reefs relative to (A) degraded reefs and (B) reference reefs.

**
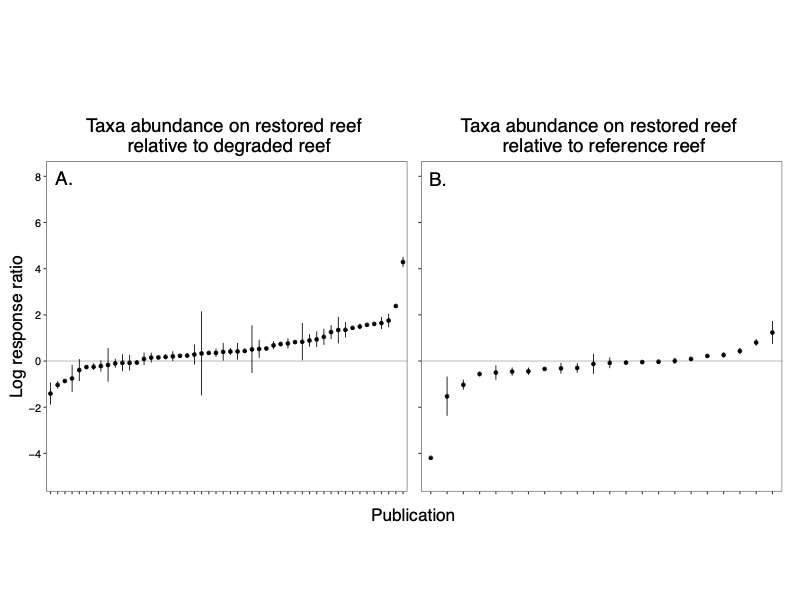
Appendix S14:** Caterpillar plots of mean log response ratio ± standard error for each publication for community taxa diversity on restored reefs relative to (A) degraded reefs and (B) reference reefs.

**
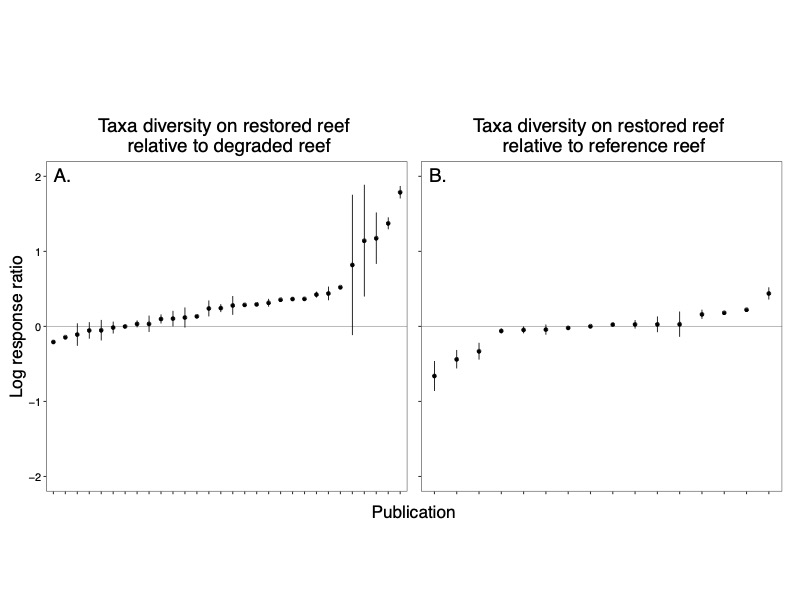
Appendix S15:** Caterpillar plots of mean log response ratio ± standard error for each publication for nitrogen removal on restored reefs relative to degraded reefs.

**
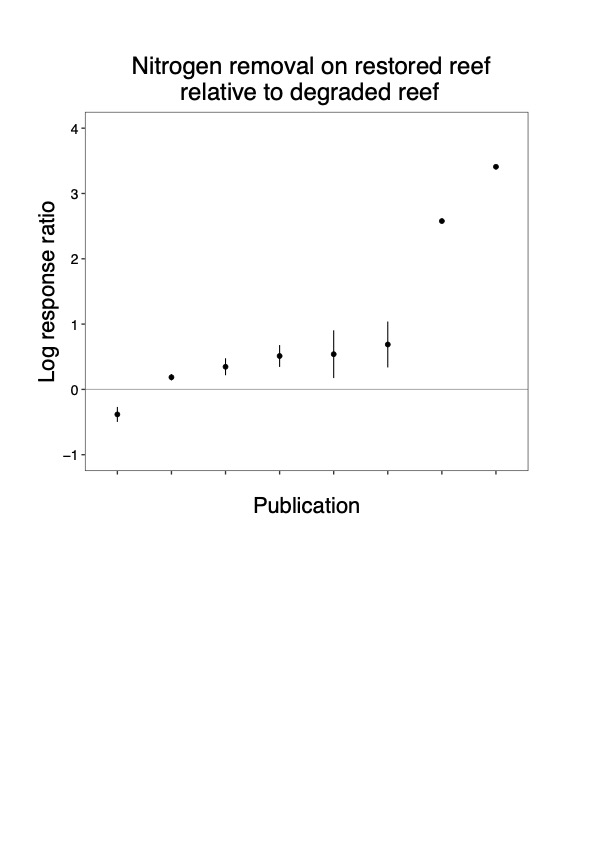
**

**Appendix S16:** Caterpillar plots of mean log response ratio ± standard error for each publication for (A-B) sediment nutrients and (C-D) water column nutrients on restored reefs relative to degraded reefs and reference reefs.

**
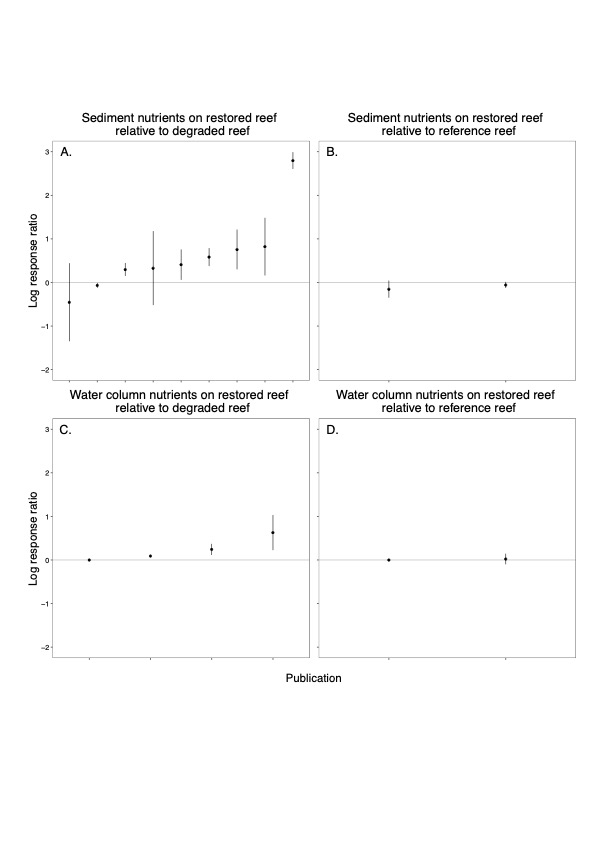
**

**Appendix S16:** Caterpillar plots of mean log response ratio ± standard error for each publication for (A-B) sediment organic matter and (C-D) water column organic matter on restored reefs relative to degraded reefs and reference reefs.

**
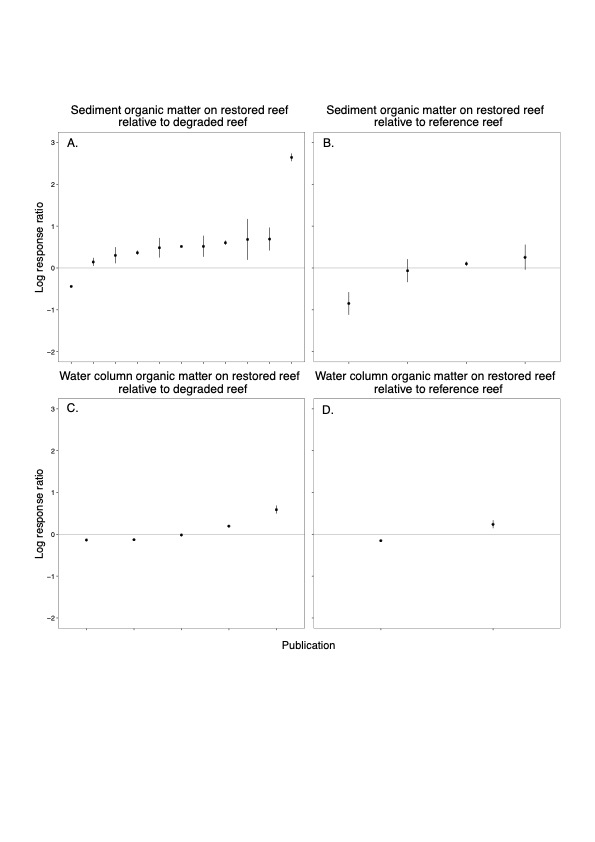
**

**Appendix S17:** Caterpillar plots of mean log response ratio ± standard error for each publication for water clarity on restored reefs relative to (A) degraded reefs and (B) reference reefs.

**
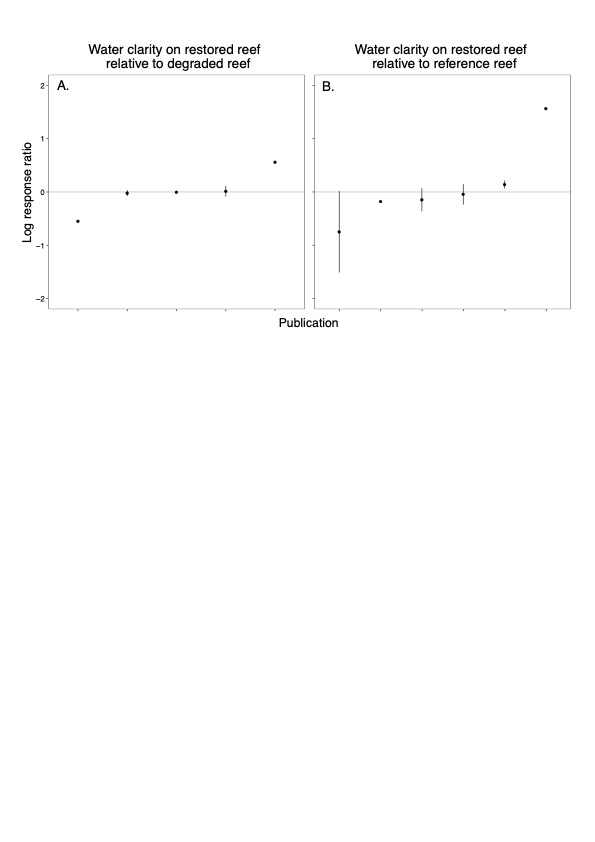
**

**Appendix S18:** Caterpillar plots of mean log response ratio ± standard error for each publication for dampened hydrodynamics on restored reefs relative to (A) degraded reefs and (B) reference reefs and for (C) shoreline advance on restored reefs relative to degraded reefs.


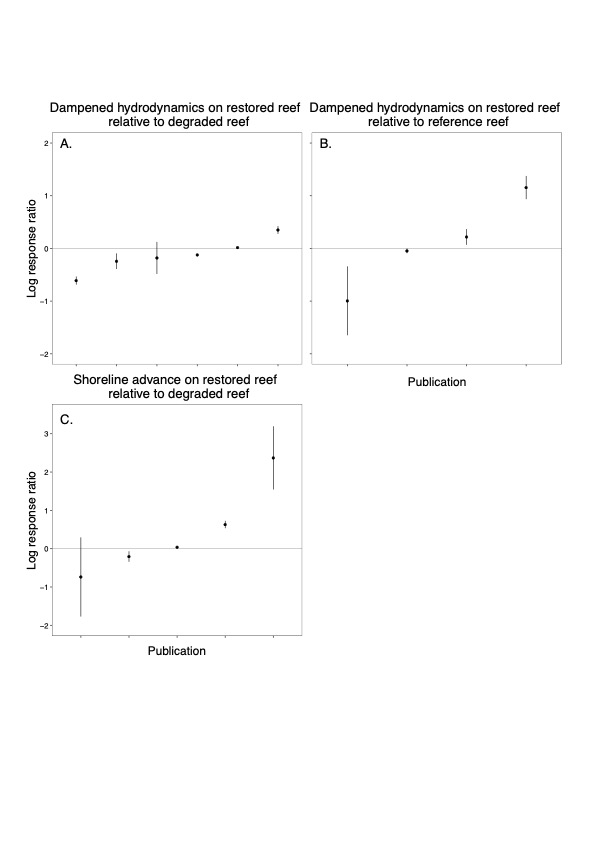


**Appendix S19:** Oyster abundance on restored reefs relative to degraded and reference reefs for (A) variance-weighted log response ratios, (B) sample size-weighted log response ratios, and (C) Hedges’ *d*. Equally-weighted means from the full dataset are reproduced in grey unfilled circles in (A) and (B) (from Figure 4, main text). Points represent mean values and error bars show 95% confidence intervals. The number of papers and effect sizes (parenthetically) are shown next to each mean effect size.

**
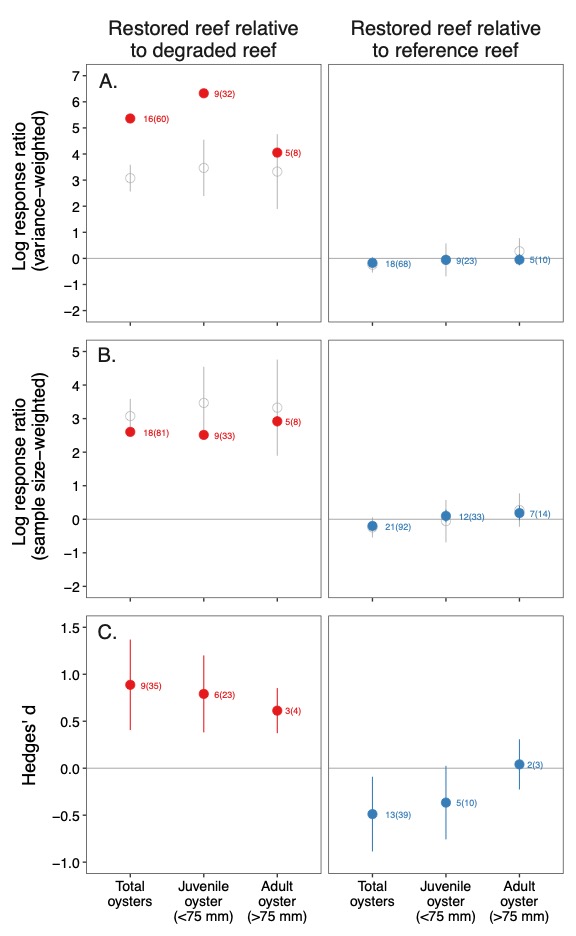
**

**Appendix S20:** Community abundance, richness, length and biomass on restored reefs relative to degraded and reference reefs for (A) variance-weighted log response ratios, (B) sample size-weighted log response ratios, and (C) Hedges’ *d*. Equally-weighted means from the full dataset are reproduced in grey unfilled circles in (A) and (B) (from Figure 5, main text). Points, error bars, asterisks, and numbers as in Appendix S19. ‘I. D.’ indicates insufficient data for analysis (< 3 publications).

**
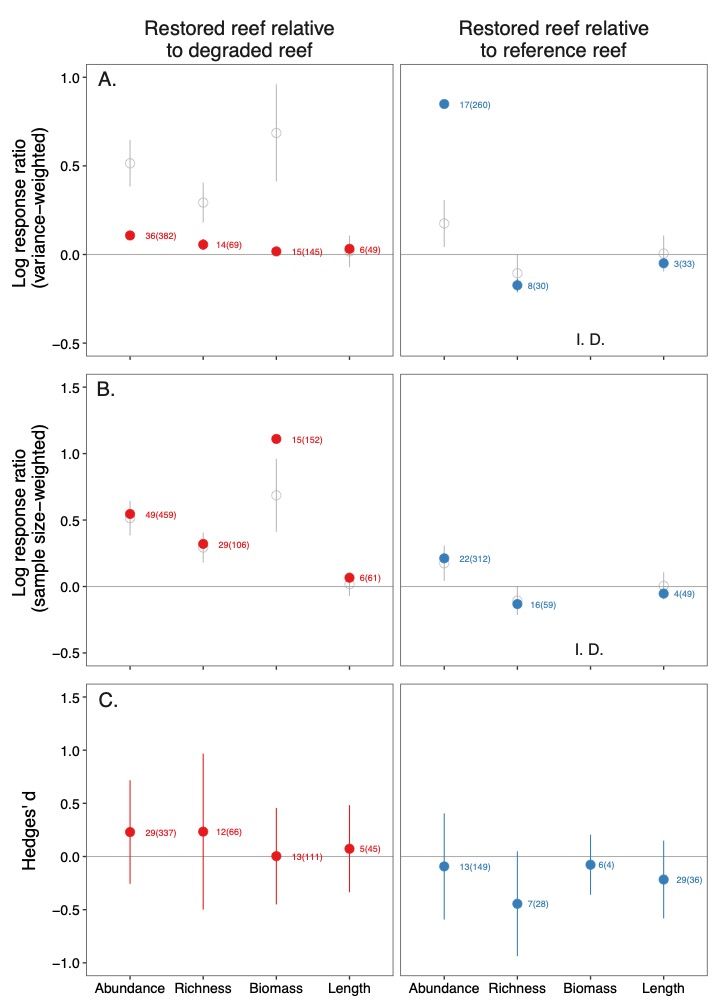
**

**Appendix S21:** Taxa group abundance for nekton, epifauna, infauna, birds, and plants on restored reefs relative to degraded and reference reefs for (A) variance-weighted log response ratios, (B) sample size-weighted log response ratios, and (C) Hedges’ *d*. Equally-weighted means from the full dataset are reproduced in grey unfilled circles in (A) and (B) (from Figure 5, main text). Points, error bars, asterisks, and numbers as in Appendix S19. ‘I. D.’ indicates insufficient data for analysis (< 3 publications).

**
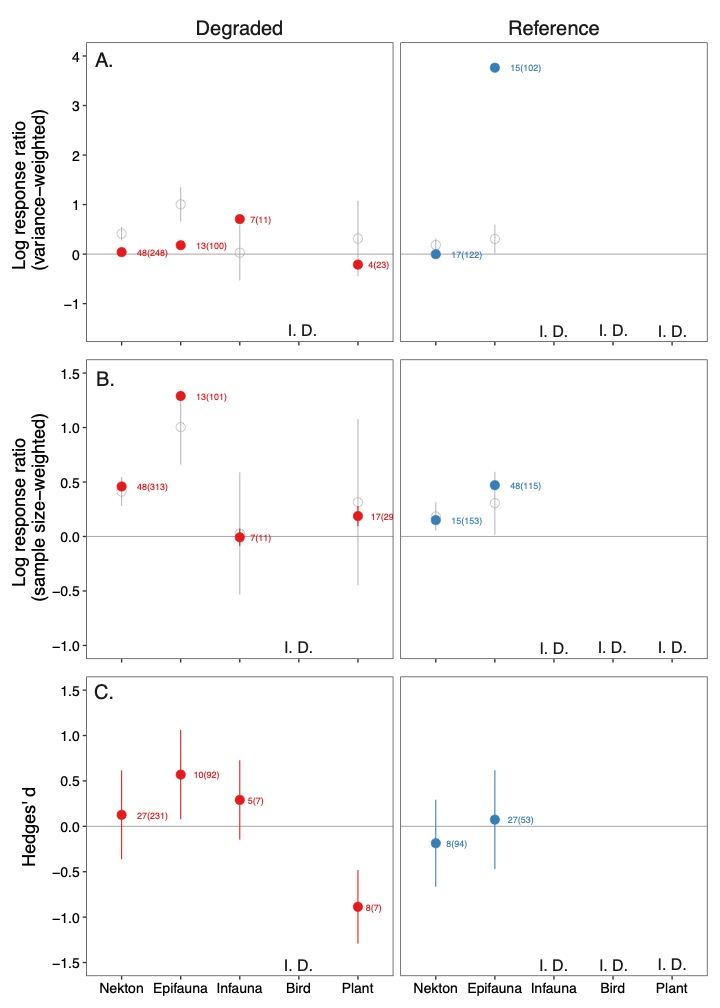
**

**Appendix S21:** (A) Nitrogen removal, (B) sediment and water column nutrients, (C) organic matter, and (D) chlorophyll concentrations, (E) water clarity, and (F) shoreline protection on restored reefs relative to degraded and reference reefs for variance-weighted log response ratios. Equally-weighted means from the full dataset are reproduced in grey unfilled circles (from Figure 6, main text). Points, error bars, asterisks, and numbers as in Appendix S19. ‘I. D.’ indicates insufficient data for analysis (< 2 publications).

**
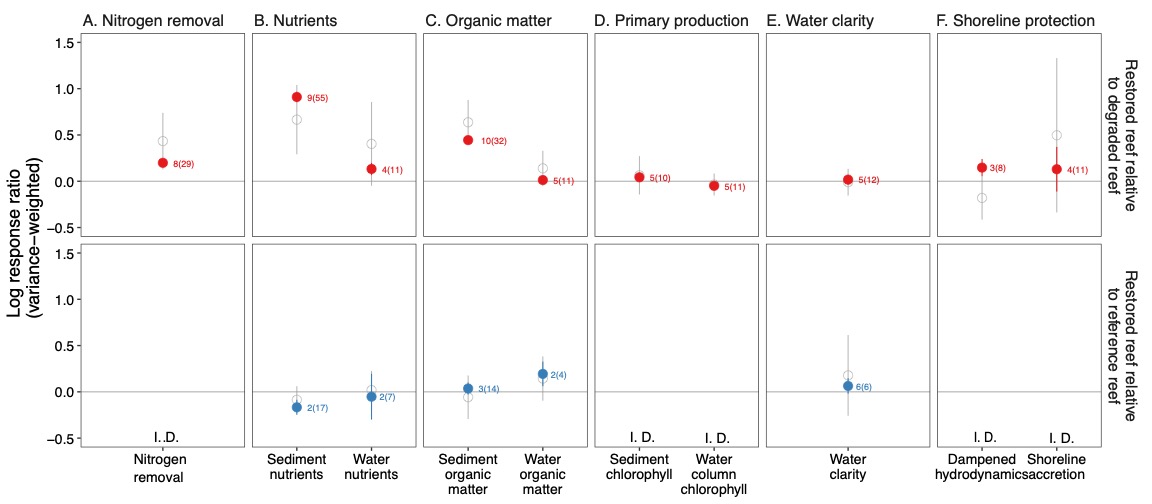
**

**Appendix S22:** (A) Nitrogen removal, (B) sediment and water column nutrients, (C) organic matter, and (D) chlorophyll concentrations, (E) water clarity, and (F) shoreline protection on restored reefs relative to degraded and reference reefs for sample size-weighted log response ratios. Equally-weighted means from the full dataset are reproduced in grey unfilled circles (from Figure 6, main text). Points, error bars, asterisks, and numbers as in Appendix S19. ‘I. D.’ indicates insufficient data for analysis (< 2 publications).


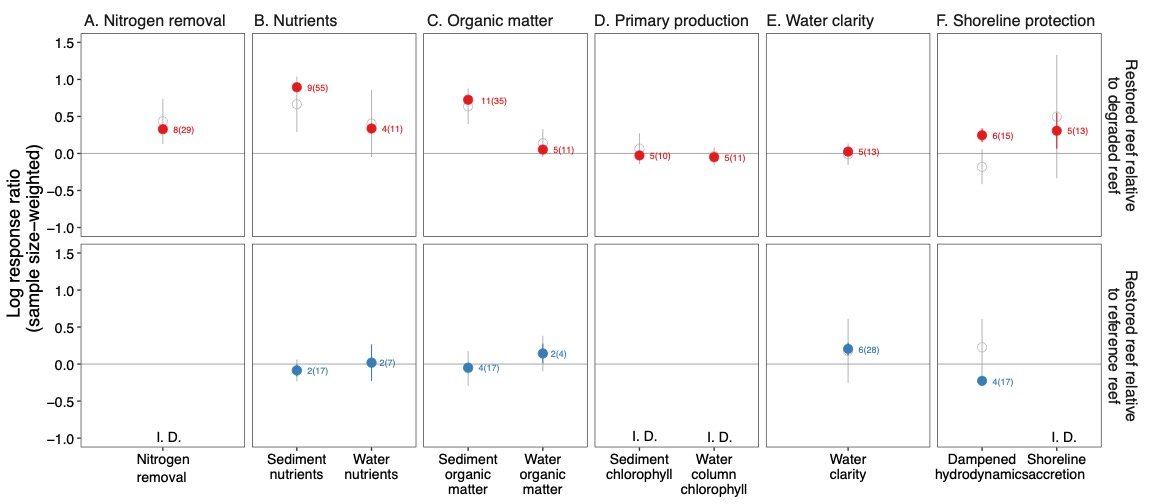


**Appendix S23:** (A) Nitrogen removal, (B) sediment and water column nutrients, (C) organic matter, and (D) chlorophyll concentrations, and (E) shoreline protection on restored reefs relative to degraded and reference reefs for Hedges’ *d*. Equally-weighted means from the full dataset are reproduced in grey unfilled circles (from Figure 6, main text). Points, error bars, asterisks, and numbers as in Appendix S19. ‘I. D.’ indicates insufficient data for analysis (< 2 publications).

**
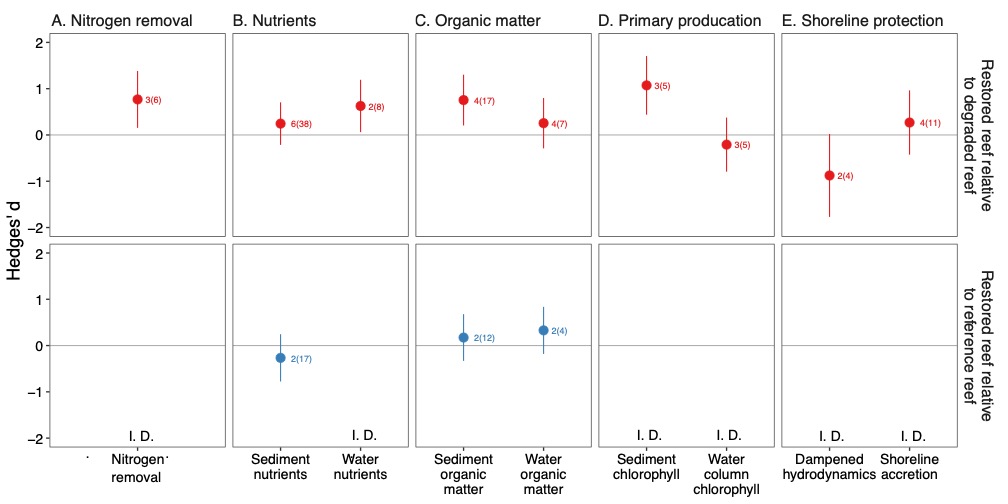
**

**References:**

Hedges LV, Olkin I. 1984. Nonparametric estimators of effect size in meta-analysis. Psychological Bulletin 96: 573–580.

Lefcheck JS, Hughes BB, Johnson AJ, Pfirrmann BW, Rasher DB, Smyth AR, Williams BL, Beck MW, Orth RJ. 2019. Are coastal habitats important nurseries? A meta-analysis. Conservation Letters 75: e12645-12.

Rosenthal R. 1979. The file drawer problem and tolerance for null results. Psychological Bulletin 86: 638–641.

Moher D, Liberati A, Tetzlaff J, Altman DG, Group P. 2009. Preferred reporting items for systematic reviews and meta-analyses: the PRISMA statement. PLoS med 6:e1000097.
